# Use and effectiveness of remdesivir for the treatment of patients with covid-19 using data from the Lean European Open Survey on SARS-CoV-2 infected patients (LEOSS): a multicentre cohort study

**DOI:** 10.1007/s15010-023-01994-0

**Published:** 2023-02-10

**Authors:** Lisa Pilgram, Katharina S. Appel, Maria M. Ruethrich, Carolin E. M. Koll, Maria J. G. T. Vehreschild, Susana M. Nunes de Miranda, Martin Hower, Kerstin Hellwig, Frank Hanses, Kai Wille, Martina Haselberger, Christoph D. Spinner, Juergen Vom Dahl, Bernd Hertenstein, Timm Westhoff, J. Janne Vehreschild, Björn-Erik Ole Jensen, Melanie Stecher

**Affiliations:** 1 Department of Nephrology and Medical Intensive Care, Charité–Universitätsmedizin Berlin, Augustenburger Platz 1, 13353 Berlin, Germany; 2 Berlin Institute of Health at Charité–Universitätsmedizin Berlin, BIH Biomedical Innovation Academy, BIH Charité Junior Digital Clinician Scientist Program, Berlin, Germany; 3 Department of Internal Medicine II, University Hospital Jena, Jena, Germany; 4 Faculty of Medicine and Department I of Internal Medicine, University of Hospital Cologne, Center for Integrated Oncology Aachen Bonn Cologne Duesseldorf, Cologne, Germany; 5 German Centre for Infection Research (DZIF), Partner Site Bonn-Cologne, Cologne, Germany; 6 Department of Internal Medicine, Infectious Diseases, Goethe University Frankfurt, Frankfurt, Germany; 7 Department of Pneumology, Infectiology, Internal Medicine and Intensive Care, Klinikum Dortmund GmbH, Hospital of University Witten/Herdecke, Dortmund, Germany; 8 Department of Neurology, St. Josef-Hospital Bochum, Ruhr University Bochum, Bochum, Germany; 9 Emergency Department, University Hospital Regensburg, Regensburg, Germany; 10 Department for Infectious Diseases and Infection Control, University Hospital Regensburg, Regensburg, Germany; 11 University of Bochum, University Clinic for Hematology, Oncology, Hemostaseology and Palliative Care, Minden, Germany; 12 Department of Internal Medicine I, Hospital Passau, Passau, Germany; 13 Department of Internal Medicine II, School of Medicine, Technical University of Munich, University Hospital Rechts Der Isar, Munich, Germany; 14 Department of Cardiology, Hospital Maria Hilf GmbH Moenchengladbach, Moenchengladbach, Germany; 15 Department of Internal Medicine I, Hospital Bremen-Mitte, Bremen, Germany; 16 Department of Internal Medicine I, Marien Hospital Herne Ruhr University Bochum, Herne, Germany; 17 Department of Gastroenterology, Hepatology and Infectious Diseases, Medical Faculty, University Hospital Düsseldorf, Heinrich Heine University, Düsseldorf, Germany; 18 Hospital of University Witten, Herdecke, Germany; 19 Department of Internal Medicine, Hematology and Oncology, Goethe University Frankfurt, Frankfurt, Germany

**Keywords:** COVID-19, Pandemic, Remdesivir, SARS-CoV-2, Steroid

## Abstract

**Objectives:**

The use of remdesivir (RDV) as the first drug approved for coronavirus disease 2019 (COVID-19) remains controversial. Based on the Lean European Open Survey on severe acute respiratory syndrome coronavirus-2 (SARS-CoV-2) infected patients (LEOSS), we aim to contribute timing-focused complementary real-world insights to its evaluation.

**Methods:**

SARS-CoV-2 infected patients between January 2020 and December 2021 treated with RDV were matched 1:1 to controls considering sociodemographics, comorbidities and clinical status. Multiple imputations were used to account for missing data. Effects on fatal outcome were estimated using uni- and multivariable Cox regression models.

**Results:**

We included 9,687 patients. For those starting RDV administration in the complicated phase, Cox regression for fatal outcome showed an adjusted hazard ratio (aHR) of 0.59 (95%CI 0.41–0.83). Positive trends could be obtained for further scenarios: an aHR of 0.51 (95%CI 0.16–1.68) when RDV was initiated in uncomplicated and of 0.76 (95% CI 0.55–1.04) in a critical phase of disease. Patients receiving RDV with concomitant steroids exhibited a further reduction in aHR in both, the complicated (aHR 0.50, 95%CI 0.29–0.88) and critical phase (aHR 0.63, 95%CI 0.39–1.02).

**Conclusion:**

Our study results elucidate that RDV use, in particular when initiated in the complicated phase and accompanied by steroids is associated with improved mortality. However, given the limitations of non-randomized trials in estimating the magnitude of the benefit of an intervention, further randomized trials focusing on the timing of therapy initiation seem warranted.

**Supplementary Information:**

The online version contains supplementary material available at 10.1007/s15010-023-01994-0.

## Introduction

Since the first cases of the severe acute respiratory syndrome coronavirus 2 (SARS-CoV-2) in late 2019, Coronavirus disease 2019 (COVID-19) still remains a global public health threat challenging the healthcare systems, economies and societies worldwide [1]. The clinical course of SARS-CoV-2 infection ranges from asymptomatic to critical with the need for mechanical ventilation. As of the beginning of November 2022, more than 633.2 million confirmed infected cases with 6.5 million deaths have been reported worldwide [2]. By now more than half of the world’s population is fully vaccinated [3]. However, there is still a high number of non-immunized people, health care resources are limited and variants with immune escape [4] as reported for Omicron evolve. Thus, COVID-19-specific treatment remains an essential part of disease management [5].

Based on the aggregated knowledge, the pathogenesis of COVID-19 includes direct viral effects on many organs as well as hyperinflammation and coagulopathy as indirect effects [6]. To address these various effects, several COVID-19-specific treatment strategies have been developed in addition to support interventions. Randomized controlled trials (RCTs) have investigated the antiviral effects of several drugs (e.g. remdesivir (RDV) [7], sotrovimab [8], nirmatrelvir/ritonavir [9], molnupiravir [10], casirivimab/imdevimab [11]) as well as immunomodulatory therapies (corticosteroids [12], tocilizumab [13], baricitinib [14]).

RDV has early been approved as a COVID-19-specific treatment by medicine regulatory authorities (e.g. European Medicines Agency (EMA), U.S. Food and Drug Administration (FDA)) [15, 16]. Authorisation details as well as recommendations have been adjusted multiple times [16]. From November 2020 on, the World Health Organization (WHO) weakly recommended against the use of RDV, changed meanwhile to recommending weakly for RDV depending on disease severity [17]. In contrast, German guidelines changed in February 2021 to neither recommending against nor for the use of RDV and, as of November 2022, state a possible use in high-risk patients early in disease [18]. Uncertainty about the benefit of RDV is based on inconclusive results of the three largest RCTs namely the Adaptive COVID-19 Treatment Trial (ACTT-1) [19], the WHO Solidarity trial [20], and more recently the Trial of Treatments for COVID-19 in Hospitalized Adults (DisCoVeRy) [21].

While RCTs are considered the gold standard for proof of the efficacy of interventions, registry-based studies are able to address the actual real-world context [22, 23]. So far existing studies which address antiviral drugs, in particular RDV, are mostly based on small case numbers or without a control group [24–26]. Thus, comprehensive observational data in Germany regarding the use of COVID-19-specific treatment and its effectiveness is scarce but needed to support guidelines and clinical decision-making [27, 28].

The Lean European Open Survey on SARS-CoV-2 infected patients (LEOSS) as a transregional and -sectoral, multicentre cohort is able to contribute complementary real-world data. Based on LEOSS, we aim to describe the pattern of RDV use and evaluate its applicability and effectiveness in preventing fatal outcomes in a real-world context.

## Methods

### Study design and patient cohort

**Fig. 1:**
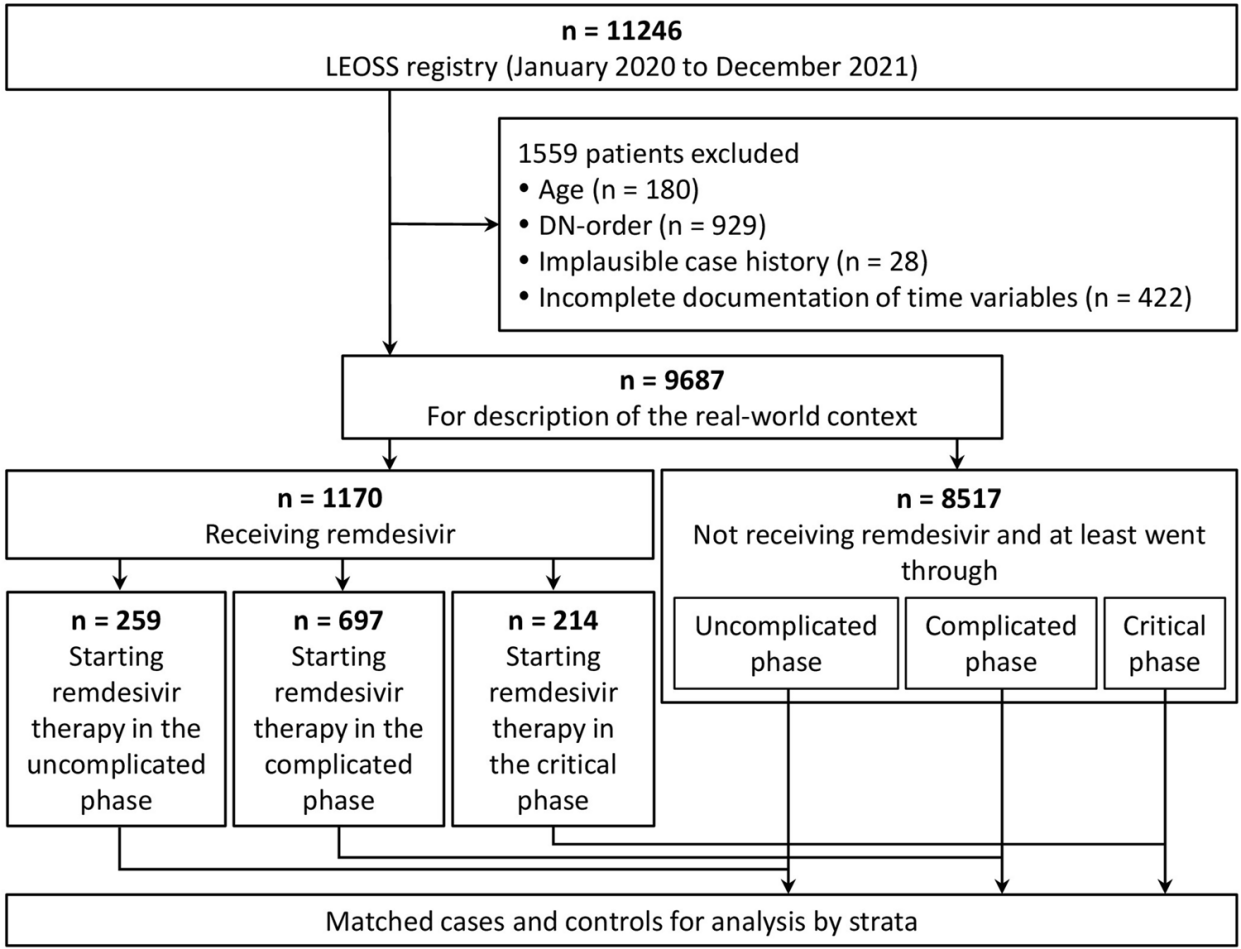
Study flow chart. Sub-setting of the cohort is performed after having applied the indicated exclusion criteria and is determined by the start of remdesivir (RDV) treatment according to the phases of the disease (figure S1). Age as an exclusion criterion was applied for patients < 18 years or patients with an unclear assignment to an age equal to or above 18 years (due to data collection manners). Controls are assigned to a sub-cohort when having passed through the respective phase. Analyses including the matching itself are performed within the sub-cohorts. Uncomplicated phase: asymptomatic or symptoms of upper respiratory tract infection, fever or gastrointestinal symptoms; complicated phase: need for oxygen supplementation, partial pressure of oxygen at room air < 70 mmHg, oxygen saturation at room air < 90%, increase of aspartate aminotransferase or alanine aminotransferase > 5 × upper limit of normal, cardiac arrythmia, pericardial effusion > 1 cm or heart failure; critical phase: need for catecholamines, life-threatening cardiac arrhythmia, mechanical ventilation (invasive or non-invasive), liver failure, quick sequential organ failure assessment ≥ 2 or acute renal failure with dialysis; DN-order: Do-Not-Intubate, Do-Not-Resuscitate-Order or refusal of ICU admission

A matched-pair analysis based on data from the LEOSS cohort (https://leoss.net/) was performed [30]. In LEOSS, patients with laboratory-confirmed SARS-CoV-2 infection are included. The dataset consisted of 11,246 patients diagnosed between January 2020 and December 2021 from 134 different sites. We excluded 13.9% (1559/11,246) patients according to the exclusion criteria illustrated in Fig. 1.

Administration of RDV at any point during the disease was the criterion for being considered as a case. We defined three sub-cohorts depending on the clinical phase when RDV therapy was initiated: uncomplicated, complicated and critical phase according to LEOSS criteria (supplementary (S) Fig. S1). The assignment to the sub-cohort happened at any time after the first positive SARS-CoV-2 detection.

### Data collection

Clinical data were collected anonymously and retrospectively in an electronic case report form (eCRF) after the medical care for the acute setting has ended. Due to the anonymous data collection no longitudinal assessment and follow-up of discharged patients were possible. This method, however, avoids problems in recruitment due to language barriers, incapacity to consent or death. The eCRF was implemented using ClinicalSurveys.net of the University Hospital of Cologne and can be accessed via an online meta-data registry [31]. Diagnostics and clinical findings were aggregated in the phases of disease and categorised in the case of metric data. The anonymisation concept has been described previously [32].

### Covariables and endpoint

The primary endpoint for the analyses was fatal outcome within the observational period which covered the acute phase of disease. In the inpatient context, the end of an acute phase of disease was marked by discharge, in the outpatient context by the lack of further medical care or a minimum observational period of 14 days or in both settings by death. In our cohort, the overall observational period covered a median time of 11 days (interquartile range (IQR) 6–19 days). Potential confounders were chosen according to the current literature. We aggregated the months of the first diagnoses to three phases of the COVID-19 pandemic in Germany: first (January–September 2020), second (October 2020–February 2021) and third (March–December 2021) phase. These phases represent time periods with similar characteristics in which patients’ management, testing strategies and disease severity did not relevantly differ between the patients.

### Statistical analysis

Data were pre-processed and analysed using R, version 4.1.0 [33]. Details and the methodological workflow are depicted in Fig. S2.

#### Missingness analyses and imputation

We performed missing analyses and applied multiple imputations on variables relevant to the analyses [34] (Table S1). The multiple imputations were carried out using the Fully Conditional Specification (FCS) method of the Multivariate Imputation by Chained Equation (MICE) algorithm in R resulting in 20 imputed data sets [35]. Detailed information on the missing data and imputation is depicted in the supplementary material (Fig. S2 and Table S13).

#### Matching strategy

Cases and controls were matched 1:1 based on exact matching on age, gender and stage at diagnosis (figure S1), and on nearest neighbour matching on further potential confounders, namely hypertension, chronic heart failure, coronary artery disease, diabetes mellitus type 2, chronic pulmonary disease, chronic kidney disease, oncological disease, body mass index (BMI), phase of the COVID-19 pandemic and use of steroids > 0.5 mg/kg prednisolone equivalent in the course of the disease. Age, gender, BMI and comorbidities were chosen as potential confounders according to current literature. We matched for stage at diagnosis and phase of the COVID-19 pandemic to account for variations in patients’ management, testing strategies and disease severity over time. Steroid administration was chosen for its demonstrated impact on the course of disease. In contrast, the efficacy of treatment with convalescent plasma and different strategies of anticoagulation has not been elucidated as comprehensively. These factors were included in the multivariable analyses to account for potential confounders. The 1:1 ratio was chosen to achieve the best-balanced composition. We applied matching methods independently in the three sub-cohorts.

#### Effect estimation

For the sub-cohorts, we used Cox proportional- hazard models stratified by RDV treatment to obtain the treatment effect. For privacy reasons, patients’ time history was recorded in an aggregated way in LEOSS (i.e. drug administration in the interval between the start and end point of a clinical phase instead of the exact date). Based on clinical expertise, the first day of a phase was equated with the start of RDV therapy in that phase. The time from the start of the respective phase to the end of the observational period was used to calculate the time-to-event. False Discovery Rate adjustment was used in case of multiple comparisons. Potential confounders which were not included as matching parameters were added as covariables via enter method. The final model was chosen based on the Akaike information criterion (AIC). We repeated the analyses distinguishing between patients with and without concomitant steroid therapy > 0.5 mg/kg prednisolone equivalents. Concomitant was defined as administration in the same phase in which RDV was initiated. Further sensitivity analyses were performed on the unimputed dataset.

#### Parameter reporting

Description of patients’ characteristics was made as absolute numbers and percentages, referred to the numbers excluding missing data. We performed statistical analyses on all 20 imputed datasets and pooled the results of the descriptive analyses and the Cox regression by Rubin’s rules and reported mean with standard deviation (SD) and (adjusted) hazard ratios ((a)HRs) with 95% confidence intervals (95%CI), respectively. Survival plots are shown for all sub-cohorts from one random dataset each with the respective log-rank *p*-value. The level of significance was set as *p* < 0.05.

## Results

### Cohort

A total of 9,687 patients were included in our analyses retrieved from LEOSS, of whom 12.1% (1170/9,687) patients have received RDV as a treatment option for COVID-19. Patients’ characteristics are shown in Table 1. The majority of patients in the RDV group were male (760/1170, 65.0%), between 46 and 85 years of age (927/1170, 79.2%) and suffering from at least one comorbidity, mostly hypertension (578/1142, 50.6%).

**Table 1:** Sociodemographic and clinical characteristics of the cohort divided by individuals treated with and without remdesivir (RDV).

Parameter	RDV treatment (*n* = 1170) % (no.)	No RDV treatment (*n* = 8517) % (no.)	*p*-value
*Age*			0.562
18–45 years	16.3 (191/1170)	21.2 (1806/8517)	
46 – 65 years	40.3 (472/1170)	34.1 (2903/8517)	
66 – 85 years	38.9 (455/1170)	37.1 (3163/8517)	
> 85 years	4.4 (52/1170)	7.6 (645/8517)	
*Gender*			0.241
Male	65.0 (760/1170)	55.9 (4763/8517)	
Female	35.0 (410/1170)	44.1 (3754/8517)	
*Comorbidities*			
Hypertension	50.6 (578/1142)	48.0 (3901/8136)	0.821
Chronic heart failure	4.7 (53/1128)	7.7 (622/8118)	0.558
Coronary heart disease	13.8 (157/1134)	13.9 (1127/8118)	1.000
Other cardiovascular disease	15.9 (180/1131)	18.9 (1535/8108)	0.709
Diabetes mellitus type 2	27.0 (301/1116)	18.9 (1502/7958)	0.233
Chronic pulmonary disease	17.8 (200/1124)	13.9 (1130/8139)	0.575
Chronic kidney disease	9.8 (110/1128)	13.9 (1130/8150)	0.498
Oncological disease	10.7 (121/1130)	11.9 (972/8162)	0.964
Chronic liver disease	2.8 (31/1129)	1.7 (140/8164)	0.962
*BMI*			0.158
< 18,5 kg/m^2^	1.1 (8/727)	2.2 (109/4906)	
18,5–24,9 kg/m^2^	21.0 (153/727)	32.6 (1600/4906)	
25–29,9 kg/m^2^	34.4 (250/727)	34.8 (1708/4906)	
> 30 kg/m^2^	43.5 (316/727)	30.4 (1489/4906)	
*Smoking history*			0.552
Active smoker	21.8 (95/435)	17.4 (621/3,571)	
Former smoker	18.9 (82/435)	15.9 (568/3571)	
Non-smoker	59.3 (258/435)	66.7 (2382/3571)	
*Pre-existing immunosuppressive therapy*			
Immunosuppressive therapy	8.1 (90/1115)	9.8 (769/7843)	0.862
*Course of SARS-CoV-2 infection*			
Fatal outcome	12.6 (147/1170)	11.8 (1001/8517)	1.000
Phase* at first positive SARS-CoV-2 detection			0.070
Uncomplicated phase	52.3 (607/1160)	71.0 (6010/8462)	
Complicated phase	40.3 (467/1160)	22.5 (1903/8462)	
Critical phase	7.4 (86/1160)	5.9 (502/8462)	
Recovery phase	0.0 (0/1160)	0.3 (23/8462)	
Dead	0.0 (0/1160)	0.3 (24/8462)	
*COVID-19 treatment*			
RDV	100.0 (1170/1170)	0.0 (0/8517)	** < 0.001**
Steroids > 0.5 mg/kg prednisolone equivalents in the course of disease	57.5 (627/1090)	85.9 (6665/7763)	** < 0.001**
Convalescent plasma	3.9 (4.6/1,170)	1.6 (134/8,517)	0.574
Other COVID-19 therapy	4.1 (48/1170)	2.0 (167/8517)	0.651
*Anticoagulants*			0.068
Therapeutic anticoagulation	34.0 (389/1143)	23.2 (1896/8164)	
Prophylactic anticoagulation	47.4 (542/1143)	45.3 (3701/8164)	
No anticoagulation	18.6 (212/1143)	31.4 (2567/8164)	
Context of SARS-CoV-2 infection			
*Time of first positive SARS-CoV-2 detection*			** < 0.001**
First phase of COVID-19 pandemic	21.2 (248/1170)	45.6 (3877/8509)	
Second phase of COVID-19 pandemic	63.9 (747/1170)	41.4 (3522/8509)	
Third phase of COVID-19 pandemic	15.0 (175/1170)	13.1 (1110/8509)	
*Country of health care facility*			0.277
Germany	99.5 (1158/1164)	96.4 (8172/8479)	
Other European countries	0.5 (6/1164)	1.9 (164/8479)	
Countries beyond Europe	0.0 (0/1164)	1.7 (143/8479)	
*Type of healthcare facility*			0.087
University hospital	75.9 (883/1164)	62.0 (5258/8474)	
Non-university hospital	24.1 (280/1164)	34.6 (2936/8474)	
Medical practice	0.0 (1/1164)	3.1 (261/8474)	
Institute	0.0 (0/1164)	0.2 (19/8474)	

Data are displayed for the original data after the application of the exclusion criteria and indicated as numbers (no.), referred to the numbers excluding missing data (details in table S13) and percentages (%). We performed a Chi2-test on the relative number of patients within each subgroup and report the p-values. First phase of the COVID-19 pandemic: January 2020-September 2020; second phase of COVID-19 pandemic: October 2020-Febuary 2021; third phase of COVID-19 pandemic: March 2021-December 2021; other COVID-19 therapy: IL-6R inhibitors, JAK-inhibitors or IL-1R inhibitors; other cardiovascular disease: aortic stenosis, AV block, carotid arterial disease, peripheral vascular disease and arterial fibrillation. *According to LEOSS (Figure S1)

With a view on precautions for use in renal and hepatic impairment, in our cohort RDV was administered in 1.9% (21/1113) patients suffering from chronic kidney disease with a glomerular filtration rate (GFR) below 30 ml/min or dialysis and in 0.3% (3/1011) patients with ALT elevations > 5 × upper limit of normal (ULN) at first SARS-CoV-2 detection.

### Treatment setting

Patients receiving RDV were predominantly treated in German healthcare facilities (1158/1164, 995%) involving universities (883/1,164, 75.9%) as well as non-university hospitals (280/1164, 24.1%). 22.1% (259/1170) initiated treatment in the uncomplicated phase (without oxygen supplementation), 59.6% (697/1170) in the complicated phase and 18.3% (214/1170) in the critical phase of the disease, predominantly defined by the need for invasive ventilation (136/213, 63.8%). The timing of RDV initiation shifted towards an earlier start in the course of infection: in the first phase of the COVID-19 pandemic 26.2% (65/248) of patients started RDV in the critical, 56.0% (139/248) in the complicated phase and 17.7% (44/248) in the uncomplicated phase of the disease; compared to 10.3% (18/175), 52.0% (91/175) and 37.7% (66/175) patients in the third phase respectively.

Clinical information on study participants of each sub-cohort is provided in our supplementary material.

### Fatal outcome in patients receiving RDV

Fatal outcomes occurred for patients receiving RDV within a median time of 18 days ( IQR 12–26 days), in controls within a median time of 12 days (IQR 6–21 days). In patients with treatment starting in the uncomplicated phase, it was reported in 2.3% (6/259) of RDV cases and 5.8% (15/259) of controls. In the sub-cohort starting in the complicated phase, 8.2% (57/697) RDV cases and 13.6% (95/696) controls deceased, in the one starting in the critical phase 39.3% (84/214) and 48.6% (104/214) respectively. Details are depicted in Table 2.

**Table 2:** Characteristics of patients with RDV therapy and matched controls stratified by phase of therapy start

Parameter	Patients undergoing					
	Uncomplicated phase*		Complicated phase*		Critical phase*	
	RDV cases (*n* = 259)	Matched controls (*n* = 259)	RDV cases (*n* = 697)	Matched controls (*n* = 697)	RDV cases (*n* = 214)	Matched controls (*n *= 214)
	% (no. ± SD)	% (no. ± SD)	% (no. ± SD)	% (no. ± SD)	% (no. ± SD)	% (no. ± SD)
*Age*						
18–45 years	22.0 (57 ± 0)	22.0 (57 ± 0)	15.3 (107 ± 0)	15.3 (107 ± 0)	12.6 (27 ± 0)	12.6 (27 ± 0)
46–65 years	34.8 (90 ± 0)	34.8 (90 ± 0)	39.7 (277 ± 0)	39.7 (277 ± 0)	49.1 (105 ± 0)	49.1 (105 ± 0)
66–85 years	37.1 (96 ± 0)	37.1 (96 ± 0)	40.3 (281 ± 0)	40.3 (281 ± 0)	36.5 (78 ± 0)	36.5 (78 ± 0)
> 85 years	6.2 (16 ± 0)	6.2 (16 ± 0)	4.6 (32 ± 0)	4.6 (32 ± 0)	1.9 (4 ± 0)	1.9 (4 ± 0)
*Gender*						
Male	56.0 (145 ± 0)	56.0 (145 ± 0)	65.9 (459 ± 0)	65.9 (459 ± 0)	72.9 (156 ± 0)	72.9 (156 ± 0)
Female	44.0 (114 ± 0)	44.0 (114 ± 0)	34.1 (238 ± 0)	34.1 (238 ± 0)	27.1 (58 ± 0)	27.1 (58 ± 0)
*Comorbidities*						
Hypertension	46.5 (121 ± 1)	47.8 (124 ± 3)	50.3 (350 ± 2)	48.4 (337 ± 7)	56.7 (121 ± 1)	58.2 (125 ± 5)
Chronic heart failure	4.4 (11 ± 1)	4.8 (12 ± 3)	4.9 (34 ± 2)	3.5 (25 ± 4)	6.7 (14 ± 1)	5.0 (11 ± 3)
Coronary heart disease	14.0 (36 ± 1)	12.5 (32 ± 3)	13.2 (92 ± 2)	10.4 (73 ± 6)	17.8 (38 ± 1)	16.3 (35 ± 3)
Other cardiovascular disease	13.8 (36 ± 1)	17.6 (46 ± 4)	17.2 (120 ± 2)	16.5 (115 ± 6)	15.4 (33 ± 2)	17.4 (37 ± 6)
Diabetes mellitus type 2	24.4 (63 ± 1)	23.8 (62 ± 4)	26.7 (186 ± 3)	25.7 (179 ± 7)	33.4 (72 ± 1)	29.0 (62 ± 4)
Chronic pulmonary disease	15.8 (41 ± 1)	15.5 (40 ± 4)	18.3 (128 ± 2)	16.2 (113 ± 6)	19.8 (42 ± 2)	17.6 (38 ± 4)
Chronic kidney disease	10.5 (27 ± 1)	10.4 (27 ± 5)	9.3 (65 ± 2)	7.8 (54 ± 5)	12.9 (28 ± 1)	11.6 (25 ± 5)
Oncological disease	9.9 (26 ± 1)	8.4 (22 ± 3)	11.6 (81 ± 2)	9.4 (65 ± 5)	8.9 (19 ± 1)	9.8 (21 ± 3)
Chronic liver disease	1.9 (5 ± 0)	3.1 (8 ± 3)	2.8 (20 ± 1)	2.2 (15 ± 3)	4.2 (9 ± 1)	4.0 (9 ± 2)
*BMI*						
< 18,5 kg/m^2^	0.4 (1 ± 1)	0.5 (1 ± 2)	1.8 (13 ± 2)	1.4 (10 ± 3)	0.1 (0 ± 1)	0.1 (0 ± 1)
18,5–24,9 kg/m^2^	24.7 (64 ± 5)	23.7 (61 ± 6)	21.5 (150 ± 7)	20.5 (143 ± 11)	18.3 (39 ± 3)	16.0 (34 ± 6)
25–29,9 kg/m^2^	41.2 (107 ± 5)	43.3 (112 ± 6)	32.4 (226 ± 7)	35.4 (247 ± 9)	32.5 (69 ± 3)	33.6 (72 ± 7)
> 30 kg/m^2^	33.7 (87 ± 6)	32.5 (84 ± 7)	44.3 (309 ± 8)	42.7 (298 ± 11)	49.1 (105 ± 3)	50.3 (108 ± 6)
*Smoking history*						
Active smoker	22.2 (58 ± 4)	17.1 (44 ± 6)	19.0 (133 ± 9)	18.2 (127 ± 9)	29.9 (64 ± 5)	23.0 (49 ± 5)
Former smoker	11.6 (30 ± 4)	14.5 (38 ± 4)	19.8 (138 ± 7)	18.4 (128 ± 9)	17.7 (38 ± 4)	24.2 (52 ± 6)
Non-smoker	66.2 (171 ± 5)	68.5 (177 ± 8)	61.2 (426 ± 11)	63.3 (442 ± 13)	12.3 (26 ± 1)	8.5 (18 ± 3)
*Pre-existing immunosuppressive therapy*						
Immunosuppressive therapy	9.7 (25 ± 2)	9.8 (25 ± 5)	7.7 (54 ± 2)	9.6 (67 ± 6)	8.2 (18 ± 1)	12.3 (26 ± 4)
*Course of SARS-CoV-2 infection*						
Fatal outcome	2.3 (6 ± 0)	5.8 (15 ± 2)	8.2 (57 ± 0)	13.6 (95 ± 5)	39.3 (84 ± 0)	48.6 (104 ± 5)
Phase* at first positive SARS-CoV-2 detection						
Uncomplicated phase	100.0 (259 ± 0)	100.0 (259 ± 0)	38.5 (268 ± 1)	38.5 (268 ± 1)	37.7 (81 ± 0)	37.7 (81 ± 0)
Complicated phase	0.0 (0 ± 0)	0.0 (0 ± 0)	61.5 (428 ± 1)	61.5 (428 ± 1)	21.2 (45 ± 0)	21.2 (45 ± 0)
Critical phase	0.0 (0 ± 0)	0.0 (0 ± 0)	0.0 (0 ± 0)	0.0 (0 ± 0)	41.1 (88 ± 0)	41.1 (88 ± 0)
Recovery phase	0.0 (0 ± 0)	0.0 (0 ± 0)	0.0 (0 ± 0)	0.0 (0 ± 0)	0.0 (0 ± 0)	0.0 (0 ± 0)
Dead	0.0 (0 ± 0)	0.0 (0 ± 0)	0.0 (0 ± 0)	0.0 (0 ± 0)	0.0 (0 ± 0)	0.0 (0 ± 0)
*COVID-19 treatment*						
Remdesivir	100.0 (259 ± 0)	0.0 (0 ± 0)	100.0 (697 ± 0)	0.0 (0 ± 0)	100.0 (214 ± 0)	0.0 (0 ± 0)
Steroids > 0.5 mg/kg prednisolone equivalents in the course of the disease	31.8 (82 ± 1)	29.8 (77 ± 3)	43.1 (300 ± 3)	39.5 (275 ± 6)	55.1 (118 ± 2)	51.6 (110 ± 4)
Convalescent plasma	1.5 (4 ± 0)	1.6 (4 ± 1)	2.4 (17 ± 0)	2.8 (19 ± 3)	11.7 (25 ± 0)	7.2 (15 ± 3)
Other COVID-19 therapy	0.4 (1 ± 0)	1.5 (4 ± 2)	2.4 (17 ± 0)	3.0 (21 ± 3)	14.0 (30 ± 0)	8.5 (18 ± 3)
*Anticoagulants*						
Therapeutic anticoagulation	27.6 (71 ± 1)	24.3 (63 ± 8)	31.8 (222 ± 2)	34.1 (238 ± 12)	48.6 (104 ± 1)	51.7 (111 ± 5)
Prophylactic anticoagulation	47.2 (122 ± 1)	48.0 (124 ± 9)	50.2 (350 ± 2)	53.7 (374 ± 14)	39.0 (84 ± 1)	39.8 (85 ± 5)
No anticoagulation	25.3 (65 ± 1)	27.7 (72 ± 6)	18.0 (126 ± 2)	12.2 (85 ± 8)	12.3 (26 ± 1)	8.5 (18 ± 3)
*Time of first positive SARS-CoV-2 detection*						
First phase of COVID-19 pandemic	17.0 (44 ± 0)	15.4 (40 ± 2)	19.9 (139 ± 0)	20.1 (140 ± 6)	30.4 (65 ± 0)	31.4 (67 ± 2)
Second phase of COVID-19 pandemic	57.5 (149 ± 0)	61.6 (160 ± 4)	67.0 (467 ± 0)	66.6 (464 ± 6)	61.2 (131 ± 0)	60.2 (129 ± 3)
Third phase of COVID-19 pandemic	25.5 (66 ± 0)	23.1 (60 ± 4)	13.1 (91 ± 0)	13.4 (93 ± 5)	8.4 (18 ± 0)	8.5 (18 ± 2)

Data are shown after multiple imputations, selection of patients undergoing the respective phase of disease and matching by age category, gender, phase at first positive SARS-CoV-2 detection, hypertension, chronic heart failure, coronary heart disease, diabetes mellitus type 2, chronic pulmonary disease, chronic kidney disease, oncological disease, body mass index (BMI) and interval of the pandemic. Distribution of factors is pooled among the imputed data and reported as a mean with a standard deviation rounded to integers. First phase of the COVID-19 pandemic: January 2020-September 2020; second phase of COVID-19 pandemic: October 2020-Febuary 2021; third phase of COVID-19 pandemic: March 2021-December 2021; other COVID-19 therapy: IL-6R inhibitors, JAK-inhibitors or IL-1R inhibitors; other cardiovascular disease: aortic stenosis, AV block, carotic arterial disease, peripheral vascular disease and arterial fibrillation. * According to LEOSS (Fig. S1)

**Fig. 2:**
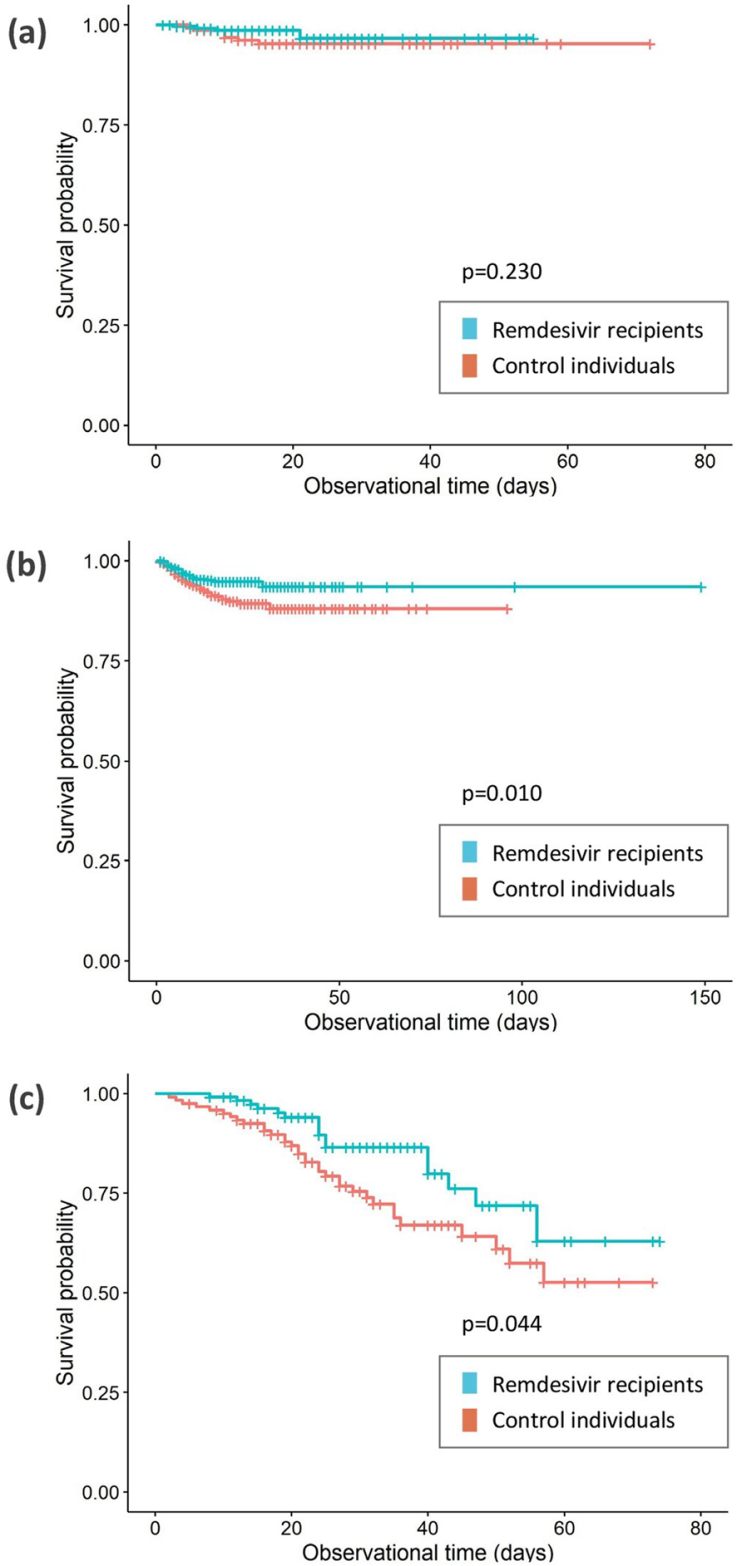
Survival plot of individuals starting remdesivir (RDV) therapy in **a** uncomplicated, **b** complicated and **c** critical phase. The observational period for each stratum starts with the beginning of the respective phase. The indicated figures are depicted from one random dataset and complemented by the *p*-value of the log-rank test. Censoring is indicated by a vertical line

In all three sub-cohorts, the (a)HR reflected a trend towards a better outcome in patients receiving RDV with a lower (a)HR the earlier in the course of SARS-CoV-2 infection the administration was initiated (Table 3; Fig. 2). The protective trend of RDV in the uni- and multivariable Cox regression achieved statistical significance only in patients starting in the complicated phase. The respective aHR of the multivariable analyses are given as 0.51 (95% CI 0.16–1.68, *p* = 0.297) starting RDV in the uncomplicated, 0.59 (95% CI 0.41–0.83, *p* = 0.004) in the complicated and 0.76 (95% CI 0.55–1.04, *p* = 0.092) in the critical phase of the disease.

**Table 3:** Cox regression model on fatal outcome of patients with RDV therapy and matched controls stratified by phase of therapy start

Parameter	Patients undergoing												
	Uncomplicated phase*				Complicated phase*				Critical phase*				
	Univariate analysis		Multivariable analysis		Univariate analysis		Multivariable analysis		Univariate analysis			Multivariable analysis	
	HR (95%-CI)	*p*-value	aHR (95%-CI)	*p*-value	HR (95%-CI)	*p*- value	aHR (95%-CI)	*p*- value		HR (95%-CI)	*p*- value	aHR (95%-CI)	*p*- value
Comorbidities**													
Other cardiovascular disease	3.54 (1.13–11.04)	0.359	3.34 (0.82–13.57)	0.139	1.50 (0.97–2.32)	0.185	1.22 (0.75–1.99)	0.434		1.44 (0.96–2.17)	0.278	1.34 (0.86–2.08)	0.197
Chronic liver disease	4.08 (0.72–23.0)	0.359	3.60 (0.49–26.44)	0.240	1.72 (0.63–4.70)	0.425	1.69 (0.59–4.85)	0.330		1.03 (0.45–2.37)	0.945	0.91 (0.39–2.11)	0.819
*Smoking history*													
Active smoker	0.62 (0.08–4.79)	0.743	0.58 (0.07–4.91)	0.633	1.13 (0.68–1.89)	0.702	1.14 (0.67–1.91)	0.636		0.88 (0.52–1.48)	0.920	0.90 (0.52–1.53)	0.690
Former smoker	1.62 (0.38–6.90)	0.674	1.06 (0.20–5.50)	0.948	1.20 (0.68–2.09)	0.670	1.13 (0.63–2.03)	0.693		0.97 (0.58–1.63)	0.945	0.88 (0.51–1.52)	0.650
Non-smoker	Ref	Ref	Ref	Ref	Ref	Ref	Ref	Ref		Ref	Ref	Ref	Ref
Pre-existing immuno-suppressive therapy**													
Immunosuppressive therapy	2.60 (0.58–11.65)	0.359	2.52 (0.42–15.02)	0.350	1.44 (0.81–2.57)	0.362	1.27 (0.70–2.29)	0.429		1.42 (0.88–2.31)	0.394	1.53 (0.92–2.54)	0.107
*Course of SARS-CoV-2 infection*													
COVID-19 treatment**													
Remdesivir	0.40 (0.14–1.13)	0.359	0.51 (0.16–1.68)	0.297	0.59 (0.42–0.83)	0.031	0.59 (0.41–0.83)	0.004		0.75 (0.55–1.03)	0.278	0.76 (0.55–1.04)	0.092
Convalescent plasma	n.a	n.a	n.a	n.a	2.25 (1.01–5.05)	0.185	2.07 (0.92–4.66)	0.084		1.05 (0.61–1.82)	0.945	0.99 (0.55–1.76)	0.967
Other COVID-19 therapy	6.54 (0.50–86.45)	0.359	7.88 (0.19–322.9)	0.329	1.22 (0.44–3.40)	0.704	1.00 (0.35–2.86)	0.999		0.76 (0.45–1.30)	0.576	0.88 (0.51–1.52)	0.656
*Anticoagulants*													
Therapeutic anticoagulation	2.55 (0.60–10.83)	0.359	2.26 (0.46–11.21)	0.347	1.43 (0.85–2.42)	0.362	1.30 (0.75–2.24)	0.357		0.79 (0.48–1.29)	0.576	0.77 (0.46–1.28)	0.317
Prophylactic anticoagulation	1.02 (0.23–4.54)	0.977	1.17 (0.19–7.08)	0.866	0.61 (0.35–1.03)	0.185	0.58 (0.34–1.00)	0.055		0.52 (0.30–0.90)	0.204	0.51 (0.29–0.89)	0.020
No anticoagulation	Ref	Ref	Ref	Ref	Ref	Ref	Ref	Ref		Ref	Ref	Ref	Ref

Data are shown after multiple imputations, selection of patients undergoing the respective phase of disease and matching by age category, gender, phase at first positive SARS-CoV-2 detection, hypertension, chronic heart failure, coronary heart disease, diabetes mellitus type 2, chronic pulmonary disease, chronic kidney disease, oncological disease, body mass index (BMI), interval of the pandemic and use of steroids in the course of the disease. *P* value of univariable analysis after correction for multiple comparisons. Other COVID-19 therapy: IL-6R inhibitors, JAK-inhibitors or IL-1R inhibitors; other cardiovascular disease: aortic stenosis, AV block, carotid arterial disease, peripheral vascular disease and arterial fibrillation. n.a.: excluded due to model quality. *According to LEOSS (Figure S1). **No reference indicated in binary variables

Prophylactic anticoagulation in patients of the complicated (aHR 0.58, 95% CI 0.34–1.00, *p* = 0.055) or critical (aHR 0.51, 95% CI 0.29–0.89, *p* = 0.020) sub-cohort exhibited an additional benefit being significant in the latter.

Sub-analyses distinguishing between males and females as well as between different age groups in the cohort starting in the complicated phase confirmed the overall trend toward a protective effect of RDV with the lowest aHR of 0.17 (95% CI 0.03–1.01, *p* = 0.360) for elderly > 85 years. Results are depicted in Tables S1-S6 in detail.

### Additional steroid administration

While having included administration of steroids > 0.5 mg/kg prednisolone equivalents throughout the course of disease as a matching parameter in the above-described cox regression, we further performed sub-analyses distinguishing between patients with and without concomitant administration of steroids in the complicated and critical starting phase of RDV. The trend toward a protective effect of RDV was sustained with a further reduction in the group receiving both RDV and prednisolone to an aHR of 0.50 (95% CI 0.29–0.88, *p* = 0.021) for those starting in the complicated phase (Table 4 and Table S7) and to an aHR of 0.63 (95% CI 0.39–1.02, *p* = 0.066) for those starting in critical phase (Table S8 and Table S9) compared to those receiving no concomitant steroid therapy.

**Table 4:** Characteristics and Cox regression model on the fatal outcome of patients starting RDV therapy in complicated phase with steroid administration and matched controls

Parameter	Patients undergoing complicated phase*					
	RDV cases (*n* = 260)	Matched controls (*n* = 260)	Univariate analysis		Multivariable analysis	
	% (no. ± SD)	% (no. ± SD)	HR (95%-CI)	*p*-value	aHR (95%-CI)	*p*-value
*Age*						
18–45 years	15.4 (40 ± 0)	15.4 (40 ± 0)	***	***	***	***
46–65 years	36.5 (95 ± 0)	36.5 (95 ± 0)	***	***	***	***
66–85 years	41.9 (109 ± 0)	41.9 (109 ± 0	***	***	***	***
> 85 years	6.2 (16 ± 0)	6.2 (16 ± 0)	***	***	***	***
*Gender*						
Male	66.5 (173 ± 0)	66.5 (173 ± 0)	***	***	***	***
Female	33.5 (87 ± 0)	33.5 (87 ± 0)	***	***	***	***
Comorbidities**						
Hypertension	49.9 (130 ± 1)	48.1 (125 ± 5)	***	***	***	***
Chronic heart failure	5.6 (15 ± 1)	5.9 (15 ± 3)	***	***	***	***
Coronary heart disease	17.7 (46 ± 1)	20.3 (53 ± 4)	***	***	***	***
Other cardiovascular disease	18.0 (47 ± 1)	21.0 (55 ± 4)	1.47 (0.78–2.76)	0.789	1.16 (0.59–2.26)	0.674
Diabetes mellitus type 2	27.8 (72 ± 1)	29.1 (76 ± 4)	***	***	***	***
Chronic pulmonary disease	20.5 (53 ± 1)	18.3 (48 ± 5)	***	***	***	***
Chronic kidney disease	9.0 (23 ± 1)	10.2 (26 ± 3)	***	***	***	***
Oncological disease	14.5 (38 ± 1)	12.0 (31 ± 4)	***	***	***	***
Chronic liver disease	4.0 (10 ± 1)	1.9 (5 ± 1)	1.30 (0.28–6.05)	0.977	1.50 (0.30–7.66)	0.627
*BMI*						
< 18,5 kg/m^2^	2.0 (5 ± 1)	2.3 (6 ± 2)	***	***	***	***
18,5–24,9 kg/m^2^	23.7 (62 ± 4)	23.2 (60 ± 6)	***	***	***	***
25–29,9 kg/m^2^	31.9 (83 ± 5)	32.8 (85 ± 8)	***	***	***	***
> 30 kg/m^2^	42.4 (110 ± 4)	41.8 (109 ± 7)	***	***	***	***
*Smoking history*						
Active smoker	21.7 (57 ± 5)	18.6 (48 ± 7)	1.10 (0.55–2.20)	0.977	1.12 (0.56–2.27)	0.747
Former smoker	20.6 (53 ± 5)	18.9 (49 ± 5)	1.02 (0.40–2.63)	0.977	0.98 (0.38–2.55)	0.968
Non-smoker	57.7 (150 ± 7)	62.5 (162 ± 7)	Ref	Ref	Ref	Ref
Pre-existing immunosuppressive therapy**						
Immunosuppressive therapy	7.2 (19 ± 1)	9.2 (24 ± 3)	1.56 (0.66–3.65)	0.789	1.48 (0.61–3.59)	0.392
*Course of SARS-CoV-2 infection*						
Fatal outcome	8.8 (23 ± 0)	16.9 (44 ± 4)				
Phase* at first positive SARS-CoV-2 detection						
Uncomplicated phase	40.8 (106 ± 0)	40.8 (106 ± 0)	***	***	***	***
Complicated phase	59.2 (154 ± 0)	59.2 (154 ± 0)	***	***	***	***
Critical phase	0.0 (0 ± 0)	0.0 (0 ± 0)	***	***	***	***
Recovery phase	0.0 (0 ± 0)	0.0 (0 ± 0)	***	***	***	***
Dead	0.0 (0 ± 0)	0.0 (0 ± 0)	***	***	***	***
COVID-19 treatment**						
Remdesivir	100.0 (260 ± 0)	0.0 (0 ± 0)	0.50 (0.29–0.87)	0.182	0.50 (0.29–0.88)	**0.021**
Steroids > 0.5 mg/kg prednisolone equivalents in the critical phase of disease	17.0 (44 ± 0)	12.6 (32 ± 3)	***	***	***	***
Convalescent plasma	1.1 (3 ± 0)	1.2 (3 ± 1)	1.52 (0.21–11.1)	0.977	1.40 (0.18–11.11)	0.753
Other COVID-19 therapy	3.9 (10 ± 0)	2.1 (6 ± 2)	1.03 (0.17–6.16)	0.977	1.02 (0.15–6.85)	0.983
*Anticoagulants*						
Therapeutic anticoagulation	34.0 (88 ± 1)	35.3 (92 ± 5)	1.29 (0.56–2.97)	0.977	1.36 (0.57–3.27)	0.494
Prophylactic anticoagulation	56.3 (146 ± 1)	49.8 (130 ± 6)	0.60 (0.26–1.42)	0.789	0.68 (0.28–1.62)	0.384
No anticoagulation	9.7 (25 ± 0)	14.9 (39 ± 5)	Ref	Ref	Ref	Ref
*Time of first positive SARS-CoV-2 detection*						
First phase of COVID-19 pandemic	12.7 (33 ± 0)	13.2 (34 ± 3)	***	***	***	***
Second phase of COVID-19 pandemic	67.3 (175 ± 0)	66.3 (172 ± 4)	***	***	***	***
Third phase of COVID-19 pandemic	20.0 (52 ± 0)	20.5 (53 ± 3)	***	***	***	***

Data are shown after multiple imputations, selection of patients undergoing the complicated phase of disease and matching by age category, gender, phase at first positive SARS-CoV-2 detection, hypertension, chronic heart failure, coronary heart disease, diabetes mellitus type 2, chronic pulmonary disease, chronic kidney disease, oncological disease, body mass index (BMI), interval of the pandemic and use of steroids in the critical phase of the disease. Distribution of factors is pooled among the imputed data and reported as a mean with a standard deviation rounded to integers. P value of univariable analysis after correction for multiple comparisons. First phase of the COVID-19 pandemic: January 2020-September 2020; second phase of COVID-19 pandemic: October 2020-Febuary 2021; third phase of COVID-19 pandemic: March 2021-December 2021; other COVID-19 therapy: IL-6R inhibitors, JAK-inhibitors or IL-1R inhibitors; other cardiovascular disease: aortic stenosis, AV block, carotic arterial disease, peripheral vascular disease and arterial fibrillation. n.a.: excluded due to model quality. *According to LEOSS (Figure S1). **No reference indicated in binary variables. ***Matching variables excluded from regression analysis

### Sensitivity analyses

Patients’ characteristics for both patients receiving RDV and their matched controls of the original dataset before multiple imputations are shown in Table S10, S11 and S12 with therapy starting in uncomplicated, complicated and critical phases, respectively. Sensitivity analyses on these datasets confirmed the above-indicated findings with again strong evidence of reduced risk for fatal outcome in patients initiating therapy in the complicated phase of the disease (aHR 0.38, 95% CI 0.20–0.70, *p* = 0.002).

## Discussion

According to our findings from the LEOSS cohort, RDV was mostly used in line with the approval by the EMA and further (inter)national guidelines. In particular, healthcare providers did comply with warnings for severe kidney and liver impairment as already previously reported [16, 29]. However, there was still a substantial share of early (patients with no need for oxygen supplementation) and late (in mechanically ventilated patients) treatment. These administrations took place almost entirely before the recent adjustments in the marketing authorisation. Thus, they represent most likely off-label use and reflect the scientific controversy based on the contradictory results of clinical trials. The latter leads to different recommendations [17, 18], and authorisation conditions, e.g. the broad approval for hospitalized patients regardless of disease severity by the FDA [36].

Large RCTs have so far not been able to present conclusive results for patients hospitalized with COVID-19 which made medical societies hesitate in broadly recommending RDV as a treatment option. The final report of ACTT-1 shows an overall positive effect of RDV for time to recovery as their main measure of effectiveness [19]. The Solidarity Trial did not detect significant changes neither in mortality nor in time to discharge [20], which led to restrictive adjustments in RDV guidelines [17, 18]. Results from DisCoVeRy reported delayed advanced respiratory support measures in patients receiving RDV but evidence for a generally better clinical outcome was lacking [21]. Comparable real-world data of the COVID Precision Medicine Analytics Platform Registry (JH-CROWN) confirmed ACTT-1 results with a shorter time to clinical improvement. Similar to Solidarity or DisCoVeRy, there was no significant trend towards a better outcome [37]. However, most studies were conducted with a broad approach including moderate to severe pneumonia without distinguishing specific sub-cohorts [20, 38, 39]. As displayed in a Cochrane review, data were insufficient to draw conclusions regarding the effectiveness of RDV in subgroups differentiated by their clinical phase at baseline [7]. This is of particular interest because, as with other antiviral therapeutic strategies for COVID-19 (e.g., monoclonal antibodies), the effect of RDV is most likely highly dependent on the timing of administration during the course of SARS-CoV-2 infection (direct viral effects versus immunopathogenesis).

Early in the COVID-19 pandemic, initiation of RDV in severely ill patients (requiring advanced respiratory support) was mostly uniformly reported not to be effective [19, 20]. In this advanced stage, no longer viral replication but hyperinflammation is the main cause of disease. Corticosteroids are currently the most effective COVID-19 therapy option in these patients [17]. As immunomodulatory drugs, they suppress the immune response. There have also been concerns that they may increase the risk to trigger uncontrolled viral replication as reported for the Middle East respiratory syndrome (MERS) [40]. A combination of the antiviral agent RDV with the immunomodulatory corticosteroids could potentially be useful. This was shown for the immunomodulatory drug baricitinib [41] In our study, this may explain the positive trend observed with an aHR of 0.76 (95% CI 0.55–1.04) when RDV was started in the critical phase of the disease.

In addition to hyperinflammation, coagulopathy is another important pathogenetic mechanism involved in severe COVID-19. Prophylactic anticoagulation in these patients presented as an additional beneficial factor in our analysis. This is already known in other several acute disease settings [42]. For COVID-19, prophylactic anticoagulation has been included in guidelines while the effect of therapeutic dosage remains inconclusive [43].

The efficacy of RDV in moderate disease (hospitalized patients with radiologically confirmed pneumonia) was investigated by several research groups: The final report of ACTT-1 uses time to recovery as main measure of effectiveness. It shows a positive effect of RDV. This is, in particular, true for patients who scored as five on their severity scale which is defined as hospitalized and requiring any supplemental (low-flow) oxygen [19]. A beneficial effect on clinical status was also measured by Spinner et al. but of uncertain relevance [38]. In DisCoVeRy, RDV delayed the initiation of advanced respiratory support [21]. In these studies, as well as in the cohort study conducted by Garibaldi et al. [37], there is a clear distinction between low- and high-flow oxygen administration. The effects were greater or achieved significance only in patients with low-flow oxygen administration. In the LEOSS cohort, we did not differentiate between low- and high-flow therapy. However, high-flow oxygen management generally takes place in intensive care units. Therefore, we expect our sub-cohort starting in a complicated phase to be rather on low-flow oxygen and to represent patients of category five on the WHO scale [45]. These patients benefited from a statistically significant protective effect on mortality which is in line with the findings of the mentioned studies [19, 21, 37, 42]. Although endpoints differ among the studies, patients with low-flow oxygen administration seem to be the most adequate target group for which treatment with RDV has a positive impact. Our sub-analyses could not clearly identify particularly benefiting subgroups regarding gender or age. Elderly individuals seem to be one of the most vulnerable groups considering their highest risk of death in Germany as reported by several authors [46–48]. However, due to low case numbers and a possible recruitment bias (Do-Not-Intubate, Do-Not-Resuscitate-Order or refusal of ICU admission as exclusion criterion) our results need to be interpreted with caution.

Real-world analyses based on JH-CROWN did not detect an additional effect for the combination with steroids [37]. In contrast, our results show a stronger protective trend in patients receiving concomitant corticosteroid therapy in the RDV starting phase. In this context, patients in moderate disease seem to benefit the most presenting with the lowest aHR (0.50, 95% CI 0.29–0.88) measured. These observations would be consistent with the previously mentioned hypothesis. It might in particular be true in the transition phase between the early phase characterized by viral replication to the late phase characterized by an excessive immune response. RCTs addressing these patients with concomitant steroid therapy are missing so far and should be initiated to optimize treatment options in these patients.

Supported by its antiviral mechanism and pre-clinical investigations, recent discussions pointed out the potential of RDV administration early in SARS-CoV-2 infection [49]. These considerations were confirmed by, among others, the PINETREE study where RDV decreased hospitalization rate in high-risk outpatients [50, 51]. Adjustments in the marketing authorisation and international guidelines followed [16]. The uncomplicated phase according to LEOSS criteria reflects an early stage of the disease. In our analyses, RDV initiation in this phase presents with the lowest aHR among our three sub-cohorts but without achieving statistical significance. Reasons for the inconclusive results may include limited case numbers, low occurrence of fatal outcome (2.3% (6/259) of cases versus 5.8% (15/259) of controls), the inclusion of patients regardless of their risk profile and the almost complete inpatient context of LEOSS. The latter might not represent the earliest phase of the disease. Given the mechanism by which variants escape monoclonal antibodies through escape mutations in the spike protein [52] and its demonstrated effect across all variants of concern encountered to date [53], RDV should also be considered as a potentially powerful treatment option in early disease.

While RCTs remain the gold standard to measure the efficacy of treatment options, there have been several approaches to support benefit-risk evaluation by other study designs [22]. Our analyses based on LEOSS benefit from the study’s standardized protocol, its transregional and -sectoral data collection and the large control cohort enabling advanced methodological approaches. However, there are several limitations to be indicated: (i) In LEOSS, high-granular investigations are not possible due to its anonymous character: the actual timing and length of administration, the distinction between co- and subsequent medication, the correlation with interventions (e.g. respiratory support measures) and events within one phase cannot be stated with certainty. (ii) Spinner et al. outlined that the ordinal WHO scale is based on treatment decisions rather than on clinical status. This is an important limitation as there is a wide variation in patients’ management across healthcare facilities [44]. The LEOSS phases include patient management (e.g. mechanical ventilation) as well as clinical status parameters (e.g. oxygen saturation): This might reflect the actual clinical status in a more adequate way. However, each of the three sub-cohorts still represents a broad target group not being able to account for small group-specific effects. (iii) Table 1 illustrates group differences between patients with and without RDV administration. We accounted for potential bias and included relevant variables as matching parameters or as co-variables in our regression analyses. Yet we might not have considered all possible confounding variables.

In conclusion, we found a protective effect of RDV on fatal outcomes in patients starting RDV therapy when being on low-flow oxygen therapy. This is intensified by concomitant steroids. Other initiation points (early/late in disease) exhibited a protective trend. The trend was stable within sensitivity analyses but did not achieve statistical significance. This is of particular interest for the uncomplicated phase where the magnitude of the RDV effect (aHR) is greatest. Having the limitations of non-randomized trials in mind, our results should be taken as an implication for further research. COVID-19 treatment options currently approved in the EU (dexamethasone, RDV, nirmatrelvir/ritonavir, various monoclonal antibodies, the recommendation for molnupiravir) are still limited [54] and new variants of concern emerge that limit the use of many monoclonal antibodies. Consequently, our results suggest that RDV should remain an important component of the therapeutic armamentarium if used in a well-timed manner.

## Supplementary Information

Below is the link to the electronic supplementary material.

Supplementary file1 (DOCX 601 KB)

## Data Availability

This study is based on LEOSS. Current anonymized public data from LEOSS can be obtained on the website (https://leoss.net/data/), a version have been archived online. Metadata is centrally registered 31. Access to a broad database can be requested in justified cases and needs to be discussed within the LEOSS Board of Investigators.

## References

[1] International Labour Organization (ILO). ILO Monitor: COVID-19 and the world of work. Eighth edition. Updated estimates and analysis.

[2] World Health Organization (WHO). Coronavirus (COVID-19) Dashboard, https://covid19.who.int/ (18.11.2022, date Last Accessed).

[3] Our World in Data. COVID-19 vaccinations, https://ourworldindata.org/covid-vaccinations (18.11.2022, date Last Accessed).

[4] Burki TK (2021). The race between vaccination and evolution of COVID-19 variants. Lancet Respir Med.

[5] Omer SB, Malani PN (2022). Booster vaccination to prevent COVID-19 in the era of omicron: an effective part of a layered public health approach. JAMA.

[6] Cevik M, Kuppalli K, Kindrachuk J, Peiris M (2020). Virology, transmission, and pathogenesis of SARS-CoV-2. BMJ.

[7] Ansems K et al. Remdesivir for the treatment of COVID-19. Cochrane Database Syst Rev 2021; 8: CD014962 10.1002/14651858.CD014962 10.1002/14651858.CD014962 PMC8406992 34350582

[8] Gupta A (2021). Early treatment for Covid-19 with SARS-CoV-2 neutralizing antibody sotrovimab. N Engl J Med.

[9] Hammond J (2022). Oral nirmatrelvir for high-risk, nonhospitalized adults with Covid-19. N Engl J Med.

[10] Jayk Bernal A (2021). Molnupiravir for oral treatment of Covid-19 in nonhospitalized patients. N Engl J Med.

[11] Weinreich DM (2021). REGEN-COV Antibody Combination and Outcomes in Outpatients with Covid-19. N Engl J Med.

[12] Wagner C et al. Systemic corticosteroids for the treatment of COVID-19. Cochrane Database Syst Rev 2021; 8:CD014963, 10.1002/14651858.CD014963 10.1002/14651858.CD014963 PMC8406706 34396514

[13] The Recovery Collaborative Group (2021). Tocilizumab in patients admitted to hospital with COVID-19 (RECOVERY): a randomised, controlled, open-label, platform trial. Lancet.

[14] Marconi VC (2021). Efficacy and safety of baricitinib for the treatment of hospitalised adults with COVID-19 (COV-BARRIER): a randomised, double-blind, parallel-group, placebo-controlled phase 3 trial. Lancet Respir Med.

[15] U.S. Food and Drug Administration (FDA). https://www.fda.gov/news-events/press-announcements/fda-approves-first-treatment-covid-19 (18.11.2022, Date Last Accessed).

[16] European Medicines Agency (EMA). Veklury - remdesivir, https://www.ema.europa.eu/en/medicines/human/EPAR/veklury (18.11.2022, Date Last Accessed).

[17] Rochwerg B (2020). A living WHO guideline on drugs for covid-19. BMJ.

[18] Kluge S, Janssens U et al. S2k Leitlinie – Empfehlungen zur stationären Therapie von Patienten mit COVID-19. (AWMF online. https://register.awmf.org/assets/ guidelines/113–001LGl_S3_Empfehlungen-zur-stationaeren-Therapie-von-Patienten-mit-COVID-19_2022–09_1.pdf, 18.11.2022, Date Last Accessed).

[19] Beigel JH (2020). Remdesivir for the treatment of Covid-19 — final report. N Engl J Med.

[20] WHO Solidarity Trial Consortium (2020). Repurposed antiviral drugs for Covid-19 — interim WHO solidarity trial results. N Engl J Med.

[21] Ader F (2022). Remdesivir plus standard of care versus standard of care alone for the treatment of patients admitted to hospital with COVID-19 (DisCoVeRy): a phase 3, randomised, controlled, open-label trial. Lancet Infect Dis.

[22] European Medicines Agency (EMA). Patient registries, https://www.ema.europa.eu/en/human-regulatory/post-authorisation/patient-registries (18.11.2022, Date Last Accessed).

[23] European Network for Health Technology Assessment (EUnetHTA). Vision paper on the sustainable availability of the proposed Registry Evaluation and Quality Standards Tool (REQueST). (2019).

[24] Antinori S (2020). Compassionate remdesivir treatment of severe Covid-19 pneumonia in intensive care unit (ICU) and Non-ICU patients: Clinical outcome and differences in post-treatment hospitalisation status. Pharmacol Res.

[25] Grein J (2020). Compassionate Use of Remdesivir for Patients with Severe Covid-19. N Engl J Med.

[26] Russo A et al. Efficacy of remdesivir-containing therapy in hospitalized COVID-19 patients: a prospective clinical experience. J Clin Med 2021 10.10.3390/jcm10173784 10.3390/jcm10173784 PMC8432083 34501233

[27] Lyman GH, Kuderer NM (2020). Randomized controlled trials versus real-world data in the COVID-19 era: a false narrative. Cancer Invest.

[28] Martinuka O, von Cube M, Wolkewitz M (2021). Methodological evaluation of bias in observational coronavirus disease 2019 studies on drug effectiveness. Clin Microbiol Infect.

[29] Pilgram L (2022). SARS-CoV-2 infection in chronic kidney disease patients with pre-existing dialysis: description across different pandemic intervals and effect on disease course (mortality). Infection.

[30] Pilgram L (2021). The COVID-19 pandemic as an opportunity and challenge for registries in health services research: lessons learned from the lean european open survey on sARS-CoV-2 infected patients (LEOSS). Gesundheitswesen.

[31] The LEOSS study group. LEOSS metadata on medical data models (mdm) portal., 2021. https://medical-data-models.org/42476 (18.11.2022, Date Last Accessed).

[32] Jakob CEM, Kohlmayer F, Meurers T, Vehreschild JJ, Prasser F (2020). Design and evaluation of a data anonymization pipeline to promote Open Science on COVID-19. Sci Data.

[33] R Core Team. R Foundation for Statistical Computing. (Vienna, Austria, 2020).

[34] Sterne JA (2009). Multiple imputation for missing data in epidemiological and clinical research: potential and pitfalls. BMJ.

[35] Van Buuren SG-O, Karin V, Gerko V, Schouten R et al. Multivariate Imputation by Chained Equations, https://cran.r-project.org/web/packages/ mice/mice.pdf (18.11.2022, Date Last Accessed)

[36] U.S. Food and Drug Administration (FDA). Veklury: Highlights of prescribing information. (2020).

[37] Garibaldi BT (2021). Comparison of Time to Clinical Improvement With vs Without Remdesivir Treatment in Hospitalized Patients With COVID-19. JAMA Netw Open.

[38] Goldman JD (2020). Remdesivir for 5 or 10 days in patients with severe Covid-19. N Engl J Med.

[39] Wang Y (2020). Remdesivir in adults with severe COVID-19: a randomised, double-blind, placebo-controlled, multicentre trial. Lancet.

[40] Hui DS (2018). Systemic corticosteroid therapy may delay viral clearance in patients with middle east respiratory syndrome coronavirus infection. Am J Respir Crit Care Med.

[41] Kalil AC (2021). Baricitinib plus remdesivir for hospitalized adults with Covid-19. N Engl J Med.

[42] Mismetti P (2000). Prevention of venous thromboembolism in internal medicine with unfractionated or low-molecular-weight heparins: a meta-analysis of randomised clinical trials. Thromb Haemost.

[43] Bradbury CA, McQuilten Z (2022). Anticoagulation in COVID-19. Lancet.

[44] Spinner CD (2020). Effect of remdesivir vs standard care on clinical status at 11 days in patients with moderate COVID-19: a randomized clinical trial. JAMA.

[45] World Health Organization (WHO). Novel Coronavirus COVID-19 Therapeutic Trial Synopsis. (2020).

[46] Karagiannidis C (2020). Case characteristics, resource use, and outcomes of 10 021 patients with COVID-19 admitted to 920 German hospitals: an observational study. Lancet Respir Med.

[47] Nachtigall I (2020). Clinical course and factors associated with outcomes among 1904 patients hospitalized with COVID-19 in Germany: an observational study. Clin Microbiol Infect.

[48] Raichle C (2022). Hospitalized patients dying with SARS-CoV-2 infection-An analysis of patient characteristics and management in ICU and general ward of the LEOSS registry. PLoS One.

[49] Dolken L, Stich A, Spinner CD (2021). Remdesivir for early COVID-19 treatment of high-risk individuals prior to or at early disease onset-lessons learned. Viruses.

[50] Gottlieb RL (2022). Early remdesivir to prevent progression to severe Covid-19 in outpatients. N Engl J Med.

[51] Piccicacco N (2022). Real-world effectiveness of early remdesivir and sotrovimab in the highest-risk COVID-19 outpatients during the Omicron surge. J Antimicrob Chemother.

[52] VanBlargan LA (2022). An infectious SARS-CoV-2 B.1.15.29 Omicron virus escapes neutralization by therapeutic monoclonal antibodies. Nat Med.

[53] Vangeel L (2022). Remdesivir, Molnupiravir and Nirmatrelvir remain active against SARS-CoV-2 Omicron and other variants of concern. Antiviral Res.

[54] European Medicines Agency (EMA). COVID-19 treatments, https://www.ema.europa.eu/en/human-regulatory/overview/public-health-threats/coronavirus-disease-covid-19/treatments-vaccines/covid-19-treatments (18.11.2022, Date Last Accessed).

