# Supplementary material for: Use and effectiveness of remdesivir for the treatment of patients with covid-19 using data from the Lean European Open Survey on SARS-CoV-2 infected patients (LEOSS): a multicentre cohort study
Source: Infection. 2023 Feb 10;51(4):1033–49. doi: 10.1007/s15010-023-01994-0 (PMC9913009; doi:10.1007/s15010-023-01994-0)
Supplement: Supplementary file 1 — Supplementary file1 (DOCX 601 KB) [file 15010_2023_1994_MOESM1_ESM.docx]

# Supplementary material Use and Effectiveness of Remdesivir for the Treatment of Patients with COVID-19 Using Data from the Lean European Open Survey on SARS-CoV-2 infected patients (LEOSS): A multicentre cohort study

Lisa PILGRAM^1,2,3#*^, Katharina S. APPEL^3#^, Maria M. RUETHRICH^4^, Carolin E.M. KOLL^5,6^, Maria J.G.T. VEHRESCHILD^7^, Susana M. NUNES DE MIRANDA^5^, Martin HOWER^8^, Kerstin HELLWIG^9^, Frank HANSES^10,11^, Kai WILLE^12^, Martina HASELBERGER^13^, Christoph SPINNER^14^, Juergen VOM DAHL^15^, Bernd HERTENSTEIN^16^, Timm WESTHOFF^17^, J. Janne VEHRESCHILD^3,5,6^, Björn-Erik Ole JENSEN^18#^, Melanie STECHER^5,6#^

^1^ Department of Nephrology and Medical Intensive Care, Charité - Universitätsmedizin Berlin, Berlin, Germany.

^2^ Berlin Institute of Health at Charité – Universitätsmedizin Berlin, BIH Biomedical Innovation Academy, BIH Charité Junior Digital Clinician Scientist Program, Berlin, Germany.

^3^ Department of Internal Medicine, Hematology and Oncology, Goethe University Frankfurt, Frankfurt, Germany.

^4^ Department of Internal Medicine II, University Hospital Jena, Jena, Germany.

^5^ University of Cologne, Faculty of Medicine and University Hospital Cologne, Department I of Internal Medicine, Center for Integrated Oncology Aachen Bonn Cologne Duesseldorf.

^6^ German Centre for Infection Research (DZIF), partner site Bonn-Cologne, Cologne, Germany.

^7^ Department of Internal Medicine, Infectious Diseases, Goethe University Frankfurt, Frankfurt, Germany.

^8^ Department of Pneumology, Infectiology, Internal Medicine and Intensive Care, Klinikum Dortmund GmbH, Dortmund, Hospital of University Witten / Herdecke, Germany.

^9^ Department of Neurology, St. Josef-Hospital Bochum, Ruhr University Bochum, Bochum, Germany.

^10^ Emergency Department, University Hospital Regensburg, Regensburg, Germany.

^11^ Department for Infectious Diseases and Infection Control, University Hospital Regensburg, Germany

^12^ University of Bochum, University Clinic for Hematology, Oncology, Hemostaseology and Palliative Care, Minden, Germany.

^13^ Department of Internal Medicine I, Hospital Passau, Passau, Germany.

^14^ Department of Internal Medicine II, School of Medicine, Technical University of Munich, University Hospital rechts der Isar, Munich, Germany.

^15^ Department of Cardiology, Hospital Maria Hilf GmbH Moenchengladbach, Moenchengladbach, Germany.

^16^ Department of Internal Medicine I, Hospital Bremen-Mitte, Bremen, Germany.

^17^ Department of Internal Medicine I, Marien Hospital Herne Ruhr University Bochum, Herne, Germany.

^18^ Department of Gastroenterology, Hepatology and Infectious Diseases, Medical Faculty and University Hospital Düsseldorf, Heinrich Heine University, Düsseldorf, Germany.

^#^contributed equally *corresponding author

## Clinical information on study participants

In our sub-cohort with RDV therapy start in the uncomplicated phase, patients predominantly suffered from fever (111±0/259, 42.9%), 36.7% (95±0/259) reported dry cough, 32.8% (85±0/259) dyspnoea, 12.0% (31±0/259) headache, 10.1% (26±0/259) myalgia and 9.7% (25±0/259) hyposmia/hypogeusia. Controls suffered to a similar extent from fever (111±7/258, 43.0%) and dry cough (94±8/259, 36,3%); 29.0% (74±8/255) reported dyspnoea, 12.8% (33±6/258) myalgia, 12.8% (33±6/257) headache and 10.1% (26±4/257) hyposmia/hypogeusia. No need for oxygen supplementation was present in this sub-cohort. In contrast, all (592±0/592) patients who initiated RDV in the complicated phase of disease and their controls were dependent on oxygen supplementation. Dyspnea was present in 61.5% (429±0/697) of patients receiving RDV and in 64.2% (441±6/687) of controls. Uncomplicated phase prior to RDV initiation was passed by 64.3% (448±0/697) of cases and by 62% (432±9/697) of controls. In the sub-cohort with RDV therapy start in critical phase, cases (136±0/213, 63.8 %) and controls (156±4/213, 73.2%) predominantly needed invasive ventilation. Prior to assignment to the sub-cohort of critical phase, 17.8% (38±0/214) of RDV cases had already passed uncomplicated and complicated, 29.0% (62±0/214) exclusively uncomplicated and 1.9% (4±0/214) exclusively complicated phase. Controls similarly had in part passed uncomplicated and complicated phase (33±4/214, 15.4%), 27.9% (60±4/214) had been in uncomplicated and 4.1% (9±2/214) in complicated phase only.

## Supplementary (S) figures


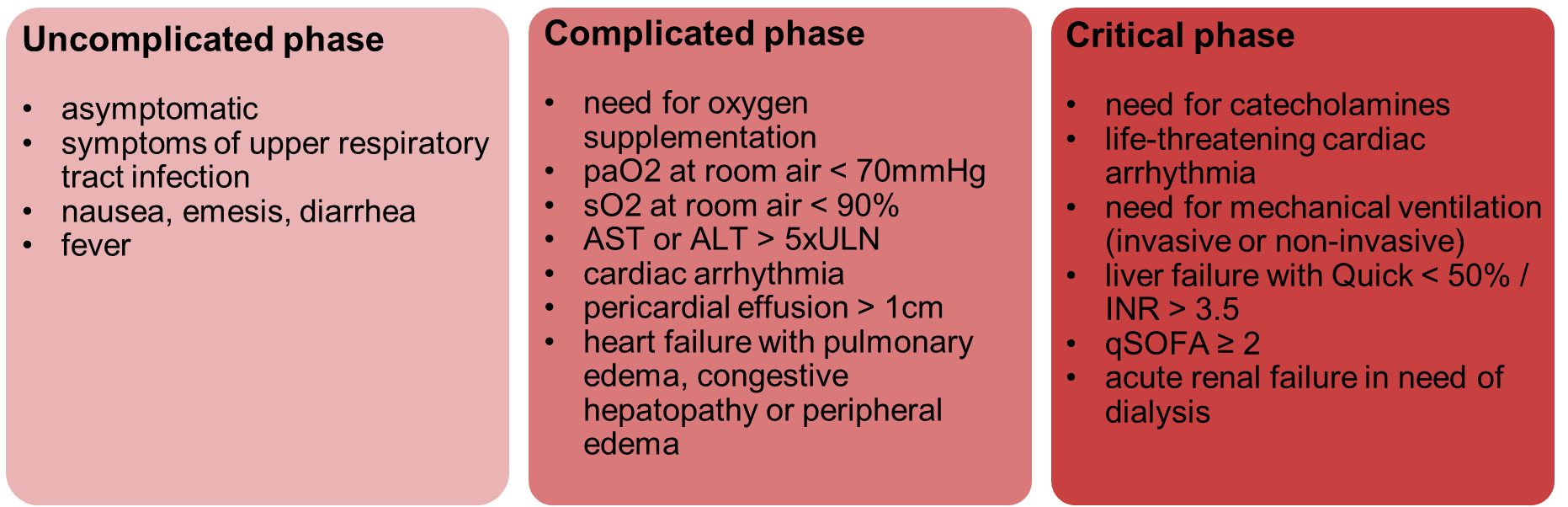


**Figure S1** Definition of phases of disease according to LEOSS. The indicated criteria (<https://leoss.net/statistics/>) only apply if occurred in the context of COVID-19. Meeting one criterion from the list even in absence of further criteria is sufficient to be assigned to the respective phase. Recovery phase is achieved when improvement by one phase or discharge. Need for oxygen supplementation is specified for prior oxygen home therapy as a clinically meaningful increase; need for mechanical ventilation for planned ventilation (e.g. due to surgery) as a prolongation > 24 hours. paO2: partial pressure of oxygen. sO2: oxygen saturation. AST: aspartate aminotransferase. ALT: alanine aminotransferase. ULN: upper limit of normal. INR: international normalized ratio. qSOFA: quick sequential organ failure assessment.

**
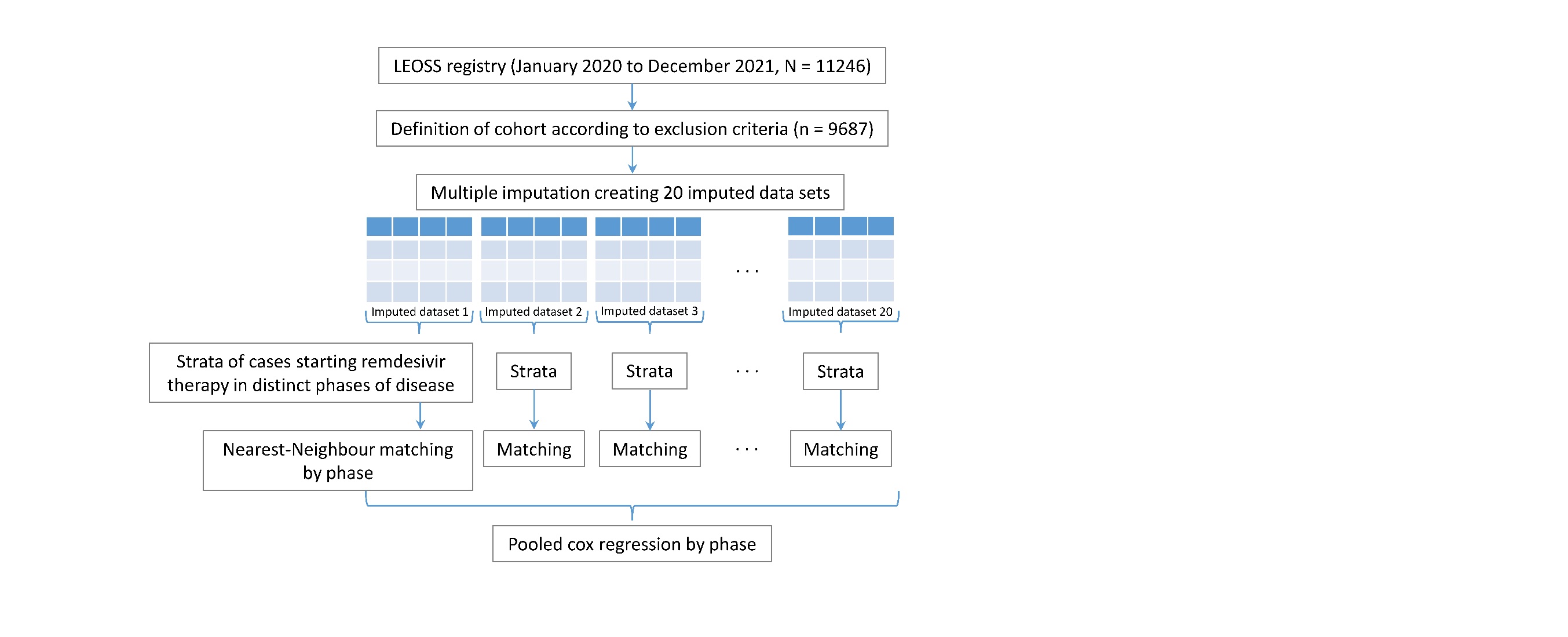
**

**Fig S2** Methodological workflow of the study. Results of missing analyses preceding multiple imputation are displayed in Table S1. Variables relevant for the analyses (Table S1) were imputed iteratively via fully conditional specification (FCS) with proportional odds model or polytomous logistic regression depending on the nature of the respective variable using the R package MICE (<https://cran.r-project.org/web/packages/mice/%20mice.pdf>). This led to a total of 20 imputed datasets. Within the three sub-cohorts, cases of each imputed dataset were matched 1:1 to corresponding controls using the R package MatchIt ([https://cran.r-project.org/web/packages/MatchIt/index. html](https://cran.r-project.org/web/packages/MatchIt/index.%20html)). Exact matching was performed on age, gender and phase at first SARS-CoV-2 detection, nearest neighbour matching on hypertension, chronic heart failure, coronary artery disease, diabetes mellitus type 2, chronic pulmonary disease, chronic kidney disease, oncological disease and BMI as well as on the phase of the pandemic and use of steroids > 0.5 mg/kg prednisolone equivalents in the course of the disease. We shuffled each imputed dataset by row to create a random selection of controls. When excluding patients with use of steroids > 0.5 mg/kg prednisolone equivalents in the starting phase of RDV, we matched on a subsequent (during critical phase) use of > 0.5 mg/kg prednisolone equivalents for cases starting RDV in the complicated phase, or dropped this matching parameter for cases starting in critical phase of disease. To assess the balance, we looked at the pre- and post-distribution of covariables, and used standardized mean differences. Effects were estimated using Cox regression and results were pooled across the 20 imputed datasets by Rubin’s rules.

## Supplementary (S) tables

**Table S1** Characteristics and Cox regression model on fatal outcome of female patients starting RDV therapy in complicated phase and matched controls.

| **Parameter** | **Patients undergoing complicated phase*** | | |  | | | | |
| --- | --- | --- | --- | --- | --- | --- | --- | --- |
|  | **RDV cases**  **(n=238)** | **Matched controls**  **(n=238)** | **Univariate analysis** | | | | **Multivariable analysis** | |
|  | **% (no.±SD)** | **% (no.±SD)** | **HR (95%-CI)** | | **p-value** | **aHR (95%-CI)** | | **p-value** |
| **Age** |  |  |  | |  |  | |  |
| 18 – 45 years | 14.7 (35±0/238) | 14.7 (35±0/238) | *** | | *** | *** | | *** |
| 46 – 65 years | 31.9 (76±0/238) | 31.9 (76±0/238) | *** | | *** | *** | | *** |
| 66 – 85 years | 44.5 (106±0/238) | 44.5 (106±0/238) | *** | | *** | *** | | *** |
| > 85 years | 8.8 (21±0/238) | 8.8 (21±0/238) | *** | | *** | *** | | *** |
| **Comorbidities**** |  |  |  | |  |  | |  |
| Hypertension | 52.9 (126±1/238) | 50.0 (119±6/238) | *** | | *** | *** | | *** |
| Chronic heart failure | 6.1 (14±1/238) | 3.8 (9±2/238) | *** | | *** | *** | | *** |
| Coronary heart disease | 9.9 (24±1/238) | 7.8 (19±3/238) | *** | | *** | *** | | *** |
| Other cardiovascular disease | 14.7 (35±1/238) | 16.2 (38±4/238) | 1.48 (0.63-3.46) | | 0.377 | 1.25 (0.48-3.24) | | 0.858 |
| Diabetes mellitus type 2 | 26.5 (63±2/238) | 24.2 (58±5/238) | *** | | *** | *** | | *** |
| Chronic pulmonary disease | 18.2 (43±1/238) | 16.9 (40±3/238) | *** | | *** | *** | | *** |
| Chronic kidney disease | 9.9 (24±1/238) | 7.6 (18±3/238) | *** | | *** | *** | | *** |
| Oncological disease | 14.0 (33±1/238) | 12.8 (31±4/238) | *** | | *** | *** | | *** |
| Chronic liver disease | 1.8 (4±0/238) | 1.3 (3±2/238) | n.a. | | n.a. | n.a. | | n.a. |
| **BMI** |  |  |  | |  |  | |  |
| < 18,5 kg/m^2^ | 2.3 (6±1/238) | 2.2 (5±2/238) | *** | | *** | *** | | *** |
| 18,5 - 24,9 kg/m^2^ | 22.5 (54±3/238) | 21.0 (50±5/238) | *** | | *** | *** | | *** |
| 25 - 29,9 kg/m^2^ | 25.8 (61±4/238) | 27.5 (65±6/238) | *** | | *** | *** | | *** |
| > 30 kg/m^2^ | 49.4 (118±3/238) | 49.4 (118±7/238) | *** | | *** | *** | | *** |
| **Smoking history** |  |  |  | |  |  | |  |
| Active smoker | 12.6 (30±5/238) | 14.4 (34±6/238) | 1.06 (0.35-3.18) | | 0.925 | 1.02 (0.33-3.11) | | 0.974 |
| Former smoker | 15.9 (38±5/238) | 10.4 (25±3/238) | 0.74 (0.20-2.76) | | 0.661 | 0.68 (0.17-2.66) | | 0.858 |
| Non-smoker | 71.6 (170±7/238) | 75.2 (179±6/238) | Ref. | | Ref. | Ref. | | Ref. |
| **Pre-existing immunosuppressive therapy**** |  |  |  | |  |  | |  |
| Immunosuppressive therapy | 10.6 (25±1/238) | 9.7 (23±4/238) | 1.67 (0.68-4.11) | | 0.270 | 1.67 (0.65-4.27) | | 0.688 |
| **Course of SARS-CoV-2 infection** |  |  |  | |  |  | |  |
| Fatal outcome | 8.0 (19±0/238) | 11.8 (28±3/238) |  | |  |  | |  |
| ***Phase* at first positive SARS-CoV-2 detection*** |  |  |  | |  |  | |  |
| Uncomplicated phase | 37.8 (90±0/238) | 37.8 (90±0/238) | *** | | *** | *** | | *** |
| Complicated phase | 62.1 (148±0/238) | 62.1 (148±0/238) | *** | | *** | *** | | *** |
| Critical phase | 0.0 (0±0/238) | 0.0 (0±0/238) | *** | | *** | *** | | *** |
| Recovery phase | 0.0 (0±0/238) | 0.0 (0±0/238) | *** | | *** | *** | | *** |
| Dead | 0.0 (0±0/238) | 0.0 (0±0/238) | *** | | *** | *** | | *** |
| ***COVID-19 treatment***** |  |  |  | |  |  | |  |
| Remdesivir | 100.0 (238±0/238) | 0.0 (0±0/238) | 0.66 (0.35-1.24) | | 0.202 | 0.63 (0.33-1.21) | | 0.624 |
| Steroids > 0.5 mg/kg prednisolone equivalents  in the course of disease | 40.6 (97±2/238) | 37.6 (90±4/238) | *** | | *** | *** | | *** |
| Convalescent plasma | 2.1 (5±0/238) | 2.2 (5±2/238) | n.a. | | n.a. | n.a. | | n.a. |
| Other COVID-19 therapy | 2.5 (6±0/238) | 2.1 (5±2/238) | n.a. | | n.a. | n.a. | | n.a. |
| ***Anticoagulants*** |  |  |  | |  |  | |  |
| Therapeutic anticoagulation | 31.9 (76±1/238) | 31.8 (76±4/238) | 0.98 (0.39-2.47) | | 0.962 | 0.85 (0.33-2.18) | | 0.858 |
| Prophylactic anticoagulation | 49.0 (116±1/238) | 56.2 (134±5/238) | 0.53 (0.20-1.39) | | 0.209 | 0.48 (0.18-1.30) | | 0.624 |
| No anticoagulation | 19.1 (46±1/238) | 12.0 (28±4/238) | Ref. | | Ref. | Ref. | | Ref. |
| **Time of first positive SARS-CoV-2 detection** |  |  |  | |  |  | |  |
| First phase of COVID-19 pandemic | 16.8 (40±0/238) | 18.1 (43±2/238) | *** | | *** | *** | | *** |
| Second phase of COVID-19 pandemic | 68.1 (162±0/238) | 65.5 (156±4/238) | *** | | *** | *** | | *** |
| Third phase of COVID-19 pandemic | 15.1 (36±0/238) | 16.4 (39±4/238) | *** | | *** | *** | | *** |

Data are shown after multiple imputation, selection of female patients undergoing the complicated phase of disease and matching by age category, phase at first positive SARS-CoV-2 detection, hypertension, chronic heart failure, coronary heart disease, diabetes mellitus type 2, chronic pulmonary disease, chronic kidney disease, oncological disease, body mass index (BMI) and interval of the pandemic. Distribution of factors is pooled among the imputed data and reported as mean with standard deviation rounded to integers. P value of univariable analysis after correction for multiple comparison. First phase of COVID-19 pandemic: January 2020-September 2020; second phase of COVID-19 pandemic: October 2020-Febuary 2021; third phase of COVID-19 pandemic: March 2021-December 2021; other COVID-19 therapy: IL-6R inhibitors, JAK-inhibitors or IL-1R inhibitors; other cardiovascular disease: aortic stenosis, AV block, carotic arterial disease, peripheral vascular disease and arterial fibrillation. n.a.: excluded due to model quality. * According to LEOSS (Figure S1).  ** No reference indicated in binary variables. *** Matching variables excluded from regression analysis.

**Table S2** Characteristics and Cox regression model on fatal outcome of male patients starting RDV therapy in complicated phase and matched controls.

| **Parameter** | **Patients undergoing complicated phase*** | | |  | | | | |
| --- | --- | --- | --- | --- | --- | --- | --- | --- |
|  | **RDV cases**  **(n=459)** | **Matched controls**  **(n=459)** | **Univariate analysis** | | | | **Multivariable analysis** | |
|  | **% (no.±SD)** | **% (no.±SD)** | **HR (95%-CI)** | | **p-value** | **aHR (95%-CI)** | | **p-value** |
| **Age** |  |  |  | |  |  | |  |
| 18 – 45 years | 15.7 (72±0/459) | 15.7 (72±0/459) | *** | | *** | *** | | *** |
| 46 – 65 years | 43.8 (201±0/459) | 43.8 (201±0/459) | *** | | *** | *** | | *** |
| 66 – 85 years | 38.1 (175±0/459) | 38.1 (175±0/459) | *** | | *** | *** | | *** |
| > 85 years | 2.4 (11±0/459) | 2.4 (11±0/459) | *** | | *** | *** | | *** |
| **Comorbidities**** |  |  |  | |  |  | |  |
| Hypertension | 48.9 (225±1/459) | 47.3 (217±7/459) | *** | | *** | *** | | *** |
| Chronic heart failure | 4.3 (20±2/459) | 3.4 (16±3/459) | *** | | *** | *** | | *** |
| Coronary heart disease | 14.9 (68±2/459) | 12.1 (55±6/459) | *** | | *** | *** | | *** |
| Other cardiovascular disease | 18.5 (85±2/459) | 17.1 (78±6/459) | 1.67 (1.03-2.72) | | 0.041 | 1.40 (0.83-2.35) | | 0.622 |
| Diabetes mellitus type 2 | 26.7 (123±2/459) | 26.0 (120±6/459) | *** | | *** | *** | | *** |
| Chronic pulmonary disease | 18.3 (84±2/459) | 16.2 (74±6/459) | *** | | *** | *** | | *** |
| Chronic kidney disease | 9.0 (41±1/459) | 7.7 (36±4/459) | *** | | *** | *** | | *** |
| Oncological disease | 10.4 (48±1/459) | 7.9 (36±4/459) | *** | | *** | *** | | *** |
| Chronic liver disease | 3.3 (15±1/459) | 2.8 (13±3/459) | 1.87 (0.62-5.65) | | 0.275 | 1.71 (0.53-5.52) | | 0.626 |
| **BMI** |  |  |  | |  |  | |  |
| < 18,5 kg/m^2^ | 1.6 (7±2/459) | 1.3 (6±2/459) | *** | | *** | *** | | *** |
| 18,5 - 24,9 kg/m^2^ | 20.9 (96±6/459) | 19.7 (90±9/459) | *** | | *** | *** | | *** |
| 25 - 29,9 kg/m^2^ | 35.8 (164±6/459) | 38.9 (178±9/459) | *** | | *** | *** | | *** |
| > 30 kg/m^2^ | 41.7 (191±7/459) | 40.2 (184±11/459) | *** | | *** | *** | | *** |
| **Smoking history** |  |  |  | |  |  | |  |
| Active smoker | 22.4 (103±7/459) | 21.4 (98±9/459) | 1.20 (0.65-2.22) | | 0.566 | 1.17 (0.62-2.20) | | 0.795 |
| Former smoker | 21.8 (100±5/459) | 21.6 (99±8/459) | 1.22 (0.65-2.29) | | 0.533 | 1.13 (0.59-2.14) | | 0.795 |
| Non-smoker | 55.8 (256±8/459) | 57.0 (261±12/459) | Ref. | | Ref. | Ref. | | Ref. |
| **Pre-existing immunosuppressive therapy**** |  |  |  | |  |  | |  |
| Immunosuppressive therapy | 6.1 (28±1/459) | 9.5 (43±5/459) | 1.41 (0.71-2.81) | | 0.328 | 1.16 (0.56-2.41) | | 0.795 |
| **Course of SARS-CoV-2 infection** |  |  |  | |  |  | |  |
| Fatal outcome | 8.3 (38±0/459) | 14.8 (68±4/459) |  | |  |  | |  |
| ***Phase* at first positive SARS-CoV-2 detection*** |  |  |  | |  |  | |  |
| Uncomplicated phase | 38.9 (178±1/459) | 38.9 (178±1/459) | *** | | *** | *** | | *** |
| Complicated phase | 61.1 (280±1/459) | 61.1 (280±1/459) | *** | | *** | *** | | *** |
| Critical phase | 0.0 (0±0/459) | 0.0 (0±0/459) | *** | | *** | *** | | *** |
| Recovery phase | 0.0 (0±0/459) | 0.0 (0±0/459) | *** | | *** | *** | | *** |
| Dead | 0.0 (0±0/459) | 0.0 (0±0/459) | *** | | *** | *** | | *** |
| ***COVID-19 treatment***** |  |  |  | |  |  | |  |
| Remdesivir | 100.0 (459±0/459) | 0.0 (0±0/459) | 0.54 (0.6-0.83) | | 0.006 | 0.56 (0.36-0.86) | | 0.095 |
| Steroids > 0.5 mg/kg prednisolone equivalents  in the course of disease | 44.4 (204±3/459) | 40.4 (185±5/459) | *** | | *** | *** | | *** |
| Convalescent plasma | 2.4 (11±0/459) | 3.2 (15±3/459) | 2.88 (1.28-6.51) | | 0.013 | 2.63 (1.10-6.29) | | 0.167 |
| Other COVID-19 therapy | 2.6 (12±0/459) | 3.3 (15±2/459) | 1.47 (0.40-5.47) | | 0.568 | 1.17 (0.29-4.69) | | 0.824 |
| ***Anticoagulants*** |  |  |  | |  |  | |  |
| Therapeutic anticoagulation | 31.8 (146±1/459) | 35.1 (161±7/459) | 1.58 (0.85-2.95) | | 0.151 | 1.40 (0.73-2.67) | | 0.622 |
| Prophylactic anticoagulation | 50.8 (233±2/459) | 53.1 (244±7/459) | 0.60 (0.30-1.22) | | 0.166 | 0.59 (0.29-1.21) | | 0.622 |
| No anticoagulation | 17.4 (80±1/459) | 11.8 (54±6/459) | Ref. | | Ref. | Ref. | | Ref. |
| **Time of first positive SARS-CoV-2 detection** |  |  |  | |  |  | |  |
| First phase of COVID-19 pandemic | 21.6 (99±0/459) | 21.3 (98±5/459) | *** | | *** | *** | | *** |
| Second phase of COVID-19 pandemic | 66.4 (305±0/459) | 66.7 (306±4/459) | *** | | *** | *** | | *** |
| Third phase of COVID-19 pandemic | 12.0 (55±0/459) | 12.0 (55±5/459) | *** | | *** | *** | | *** |

Data are shown after multiple imputation, selection of male patients undergoing the complicated phase of disease and matching by age category, phase at first positive SARS-CoV-2 detection, hypertension, chronic heart failure, coronary heart disease, diabetes mellitus type 2, chronic pulmonary disease, chronic kidney disease, oncological disease, body mass index (BMI) and interval of the pandemic. Distribution of factors is pooled among the imputed data and reported as mean with standard deviation rounded to integers. P value of univariable analysis after correction for multiple comparison. First phase of COVID-19 pandemic: January 2020-September 2020; second phase of COVID-19 pandemic: October 2020-Febuary 2021; third phase of COVID-19 pandemic: March 2021-December 2021; other COVID-19 therapy: IL-6R inhibitors, JAK-inhibitors or IL-1R inhibitors; other cardiovascular disease: aortic stenosis, AV block, carotic arterial disease, peripheral vascular disease and arterial fibrillation. n.a.: excluded due to model quality. * According to LEOSS (Figure S1).  ** No reference indicated in binary variables. *** Matching variables excluded from regression analysis.

**Table S3** Characteristics and Cox regression model on fatal outcome of patients aged 18 – 45 years starting RDV therapy in complicated phase and matched controls.

| **Parameter** | **Patients undergoing complicated phase*** | | |  | | | | |
| --- | --- | --- | --- | --- | --- | --- | --- | --- |
|  | **RDV cases**  **(n=107)** | **Matched controls**  **(n=107)** | **Univariate analysis** | | | | **Multivariable analysis** | |
|  | **% (no.±SD)** | **% (no.±SD)** | **HR (95%-CI)** | | **p-value** | **aHR (95%-CI)** | | **p-value** |
| **Gender** |  |  |  | |  |  | |  |
| Male | 67.3 (72±0/107) | 67.3 (72±0/107) | *** | | *** | *** | | *** |
| Female | 32.7 (35±0/107) | 32.7 (35±0/107) | *** | | *** | *** | | *** |
| **Comorbidities**** |  |  |  | |  |  | |  |
| Hypertension | 15.9 (17±0/107) | 14.3 (15±2/107) | *** | | *** | *** | | *** |
| Chronic heart failure | 0.9 (1±0/107) | 0.8 (1±1/107) | *** | | *** | *** | | *** |
| Coronary heart disease | 1.9 (2±0/107) | 1.1 (1±1/107) | *** | | *** | *** | | *** |
| Other cardiovascular disease | 1.9 (2±0/107) | 0.5 (1±1/107) | n.a. | | n.a. | n.a. | | n.a. |
| Diabetes mellitus type 2 | 9.7 (10±1/107) | 7.9 (8±1/107) | *** | | *** | *** | | *** |
| Chronic pulmonary disease | 9.6 (10±0/107) | 7.5 (8±2/107) | *** | | *** | *** | | *** |
| Chronic kidney disease | 1.0 (1±0/107) | 0.1 (0±0/107) | *** | | *** | *** | | *** |
| Oncological disease | 1.9 (2±0/107) | 1.2 (1±1/107) | *** | | *** | *** | | *** |
| Chronic liver disease | 2.9 (3±0/107) | 3.9 (4±1/107) | n.a. | | n.a. | n.a. | | n.a. |
| **BMI** |  |  |  | |  |  | |  |
| < 18,5 kg/m^2^ | 0.4 (0±1/107) | 0.2 (0±1/107) | *** | | *** | *** | | *** |
| 18,5 - 24,9 kg/m^2^ | 20.8 (22±2/107) | 20.0 (21±4/107) | *** | | *** | *** | | *** |
| 25 - 29,9 kg/m^2^ | 27.3 (29±3/107) | 31.7 (34±4/107) | *** | | *** | *** | | *** |
| > 30 kg/m^2^ | 51.5 (55±3/107) | 48.2 (52±5/107) | *** | | *** | *** | | *** |
| **Smoking history** |  |  |  | |  |  | |  |
| Active smoker | 19.7 (21±3/107) | 18.6 (20±4/107) | n.a. | | n.a. | n.a. | | n.a. |
| Former smoker | 8.1 (9±3/107) | 9.5 (10±3/107) | n.a. | | n.a. | n.a. | | n.a. |
| Non-smoker | 72.2 (77±4/107) | 71.9 (77±5/107) | Ref. | | Ref. | Ref. | | Ref. |
| **Pre-existing immunosuppressive therapy**** |  |  |  | |  |  | |  |
| Immunosuppressive therapy | 3.8 (4±0/107) | 11.5 (12±2/107) | n.a. | | n.a. | n.a. | | n.a. |
| **Course of SARS-CoV-2 infection** |  |  |  | |  |  | |  |
| Fatal outcome | 0.9 (1±0/107) | 2.2 (2±1/107) |  | |  |  | |  |
| ***Phase* at first positive SARS-CoV-2 detection*** |  |  |  | |  |  | |  |
| Uncomplicated phase | 43.0 (46±0/107) | 43.0 (46±0/107) | *** | | *** | *** | | *** |
| Complicated phase | 57.0 (61±0/107) | 57.0 (61±0/107) | *** | | *** | *** | | *** |
| Critical phase | 0.0 (0±0/107) | 0.0 (0±0/107) | *** | | *** | *** | | *** |
| Recovery phase | 0.0 (0±0/107) | 0.0 (0±0/107) | *** | | *** | *** | | *** |
| Dead | 0.0 (0±0/107) | 0.0 (0±0/107) | *** | | *** | *** | | *** |
| ***COVID-19 treatment***** |  |  |  | |  |  | |  |
| Remdesivir | 100.0 (107±0/107) | 0.0 (0±0/107) | 0.45 (0.39-5.19) | | 0.590 | n.a. | | n.a. |
| Steroids > 0.5 mg/kg prednisolone equivalents  in the course of disease | 38.9 (42±1/107) | 38.0 (41±3/107) | *** | | *** | *** | | *** |
| Convalescent plasma | 1.9 (2±0/107) | 4.4 (5±1/107) | n.a. | | n.a. | n.a. | | n.a. |
| Other COVID-19 therapy | 3.7 (4±0/107) | 5.8 (6±1/107) | 14.00 (1.11-177.38) | | 0.196 | n.a. | | n.a. |
| ***Anticoagulants*** |  |  |  | |  |  | |  |
| Therapeutic anticoagulation | 18.7 (20±0/107) | 19.7 (21±3/107) | n.a. | | n.a. | n.a. | | n.a. |
| Prophylactic anticoagulation | 63.6 (68±1/107) | 65.0 (70±4/107) | n.a. | | n.a. | n.a. | | n.a. |
| No anticoagulation | 17.6 (19±1/107) | 15.3 (16±2/107) | Ref. | | Ref. | Ref. | | Ref. |
| **Time of first positive SARS-CoV-2 detection** |  |  |  | |  |  | |  |
| First phase of COVID-19 pandemic | 23.4 (25±0/107) | 24.5 (26±2/107) | *** | | *** | *** | | *** |
| Second phase of COVID-19 pandemic | 56.0 (60±0/107) | 53.9 (58±2/107) | *** | | *** | *** | | *** |
| Third phase of COVID-19 pandemic | 20.6 (22±0/107) | 21.6 (23±2/107) | *** | | *** | *** | | *** |

Data are shown after multiple imputation, selection of patients aged 18-45 years undergoing the complicated phase of disease and matching by gender, phase at first positive SARS-CoV-2 detection, hypertension, chronic heart failure, coronary heart disease, diabetes mellitus type 2, chronic pulmonary disease, chronic kidney disease, oncological disease, body mass index (BMI) and interval of the pandemic. Distribution of factors is pooled among the imputed data and reported as mean with standard deviation rounded to integers. P value of univariable analysis after correction for multiple comparison. First phase of COVID-19 pandemic: January 2020-September 2020; second phase of COVID-19 pandemic: October 2020-Febuary 2021; third phase of COVID-19 pandemic: March 2021-December 2021; other COVID-19 therapy: IL-6R inhibitors, JAK-inhibitors or IL-1R inhibitors; other cardiovascular disease: aortic stenosis, AV block, carotic arterial disease, peripheral vascular disease and arterial fibrillation. n.a.: excluded due to model quality. * According to LEOSS (Figure S1).  ** No reference indicated in binary variables. *** Matching variables excluded from regression analysis.

**Table S4** Characteristics and Cox regression model on fatal outcome of patients aged 46 - 65 years starting RDV therapy in complicated phase and matched controls.

| **Parameter** | **Patients undergoing complicated phase*** | | |  | | | | |
| --- | --- | --- | --- | --- | --- | --- | --- | --- |
|  | **RDV cases**  **(n=277)** | **Matched controls**  **(n=277)** | **Univariate analysis** | | | | **Multivariable analysis** | |
|  | **% (no.±SD)** | **% (no.±SD)** | **HR (95%-CI)** | | **p-value** | **aHR (95%-CI)** | | **p-value** |
| **Gender** |  |  |  | |  |  | |  |
| Male | 72.6 (201±0/277) | 72.6 (201±0/277) | *** | | *** | *** | | *** |
| Female | 27.4 (76±0/277) | 27.4 (76±0/277) | *** | | *** | *** | | *** |
| **Comorbidities**** |  |  |  | |  |  | |  |
| Hypertension | 44.1 (122±5/277) | 46.2 (128±1/277) | *** | | *** | *** | | *** |
| Chronic heart failure | 1.8 (5±2/277) | 1.8 (5±1/277) | *** | | *** | *** | | *** |
| Coronary heart disease | 6.7 (18±3/277) | 8.5 (23±1/277) | *** | | *** | *** | | *** |
| Other cardiovascular disease | 7.3 (20±3/277) | 7.9 (22±1/277) | 1.23 (0.39-3.93) | | 0.727 | 0.79 (0.23-2.72) | | 0.885 |
| Diabetes mellitus type 2 | 27.0 (75±5/277) | 28.1 (78±2/277) | *** | | *** | *** | | *** |
| Chronic pulmonary disease | 12.8 (35±4/277) | 15.2 (42±1/277) | *** | | *** | *** | | *** |
| Chronic kidney disease | 6.5 (18±3/277) | 6.5 (18±1/277) | *** | | *** | *** | | *** |
| Oncological disease | 8.0 (22±4/277) | 10.0 (28±1/277) | *** | | *** | *** | | *** |
| Chronic liver disease | 2.6 (7±2/277) | 4.2 (12±1/277) | 2.12 (0.49-9.29) | | 0.327 | 2.03 (0.41-10.15) | | 0.615 |
| **BMI** |  |  |  | |  |  | |  |
| < 18,5 kg/m^2^ | 0.5 (1±1/277) | 0.8 (2±1/277) | *** | | *** | *** | | *** |
| 18,5 - 24,9 kg/m^2^ | 14.8 (41±4/277) | 13.8 (38±3/277) | *** | | *** | *** | | *** |
| 25 - 29,9 kg/m^2^ | 38.2 (106±7/277) | 34.5 (96±6/277) | *** | | *** | *** | | *** |
| > 30 kg/m^2^ | 46.5 (129±8/277) | 50.9 (141±5/277) | *** | | *** | *** | | *** |
| **Smoking history** |  |  |  | |  |  | |  |
| Active smoker | 19.9 (55±5/277) | 19.1 (53±5/277) | 2.17 (0.94-4.98) | | 0.082 | 2.10 (0.90-4.93) | | 0.343 |
| Former smoker | 20.1 (56±5/277) | 20.0 (55±6/277) | 1.71 (0.71-4.15) | | 0.244 | 1.48 (0.57-3.81) | | 0.615 |
| Non-smoker | 60.0 (166±7/277) | 60.8 (169±7/277) | Ref. | | Ref. | Ref. | | Ref. |
| **Pre-existing immunosuppressive therapy**** |  |  |  | |  |  | |  |
| Immunosuppressive therapy | 9.3 (26±4/277) | 10.1 (28±1/277) | 1.21 (0.45-3.24) | | 0.712 | 0.98 (0.33-2.94) | | 0.970 |
| **Course of SARS-CoV-2 infection** |  |  |  | |  |  | |  |
| Fatal outcome | 11.0 (30±3/277) | 7.2 (20±0/277) |  | |  |  | |  |
| ***Phase* at first positive SARS-CoV-2 detection*** |  |  |  | |  |  | |  |
| Uncomplicated phase | 40.6 (112±1/277) | 40.6 (112±1/277) | *** | | *** | *** | | *** |
| Complicated phase | 59.4 (164±1/277) | 59.4 (164±1/277) | *** | | *** | *** | | *** |
| Critical phase | 0.0 (0±0/277) | 0.0 (0±0/277) | *** | | *** | *** | | *** |
| Recovery phase | 0.0 (0±0/277) | 0.0 (0±0/277) | *** | | *** | *** | | *** |
| Dead | 0.0 (0±0/277) | 0.0 (0±0/277) | *** | | *** | *** | | *** |
| ***COVID-19 treatment***** |  |  |  | |  |  | |  |
| Remdesivir | 0.0 (0±0/277) | 100.0 (277±0/277) | 0.64 (0.35-1.17) | | 0.157 | 0.63 (0.34-1.16) | | 0.365 |
| Steroids > 0.5 mg/kg prednisolone equivalents  in the course of disease | 39.3 (109±6/277) | 41.4 (115±2/277) | *** | | *** | *** | | *** |
| Convalescent plasma | 3.0 (8±2/277) | 2.9 (8±0/277) | 3.95 (1.37-11.41) | | 0.016 | 3.45 (1.09-10.95) | | 0.226 |
| Other COVID-19 therapy | 2.8 (8±3/277) | 3.6 (10±0/277) | 1.75 (0.38-8.04) | | 0.475 | 0.96 (0.19-4.94) | | 0.970 |
| ***Anticoagulants*** |  |  |  | |  |  | |  |
| Therapeutic anticoagulation | 30.7 (85±6/277) | 30.7 (85±1/277) | 3.85 (1.23-12.08) | | 0.029 | 3.64 (1.13-11.66) | | 0.226 |
| Prophylactic anticoagulation | 56.9 (158±6/277) | 52.6 (146±1/277) | 0.46 (0.12-1.70) | | 0.252 | 0.43 (0.12-1.59) | | 0.434 |
| No anticoagulation | 12.4 (34±4/277) | 16.8 (46±1/277) | Ref. | | Ref. | Ref. | | Ref. |
| **Time of first positive SARS-CoV-2 detection** |  |  |  | |  |  | |  |
| First phase of COVID-19 pandemic | 20.2 (56±2/277) | 21.7 (60±0/277) | *** | | *** | *** | | *** |
| Second phase of COVID-19 pandemic | 65.4 (181±3/277) | 64.6 (179±0/277) | *** | | *** | *** | | *** |
| Third phase of COVID-19 pandemic | 14.4 (40±4/277) | 13.7 (38±0/277) | *** | | *** | *** | | *** |

Data are shown after multiple imputation, selection of patients aged 46-65 years undergoing the complicated phase of disease and matching by gender, phase at first positive SARS-CoV-2 detection, hypertension, chronic heart failure, coronary heart disease, diabetes mellitus type 2, chronic pulmonary disease, chronic kidney disease, oncological disease, body mass index (BMI) and interval of the pandemic. Distribution of factors is pooled among the imputed data and reported as mean with standard deviation rounded to integers. P value of univariable analysis after correction for multiple comparison. First phase of COVID-19 pandemic: January 2020-September 2020; second phase of COVID-19 pandemic: October 2020-Febuary 2021; third phase of COVID-19 pandemic: March 2021-December 2021; other COVID-19 therapy: IL-6R inhibitors, JAK-inhibitors or IL-1R inhibitors; other cardiovascular disease: aortic stenosis, AV block, carotic arterial disease, peripheral vascular disease and arterial fibrillation. n.a.: excluded due to model quality. * According to LEOSS (Figure S1).  ** No reference indicated in binary variables. *** Matching variables excluded from regression analysis.

**Table S5** Characteristics and Cox regression model on fatal outcome of patients aged 66 – 85 years starting RDV therapy in complicated phase and matched controls.

| **Parameter** | **Patients undergoing complicated phase*** | | |  | | | | |
| --- | --- | --- | --- | --- | --- | --- | --- | --- |
|  | **RDV cases**  **(n=281)** | **Matched controls**  **(n=281)** | **Univariate analysis** | | | | **Multivariable analysis** | |
|  | **% (no.±SD)** | **% (no.±SD)** | **HR (95%-CI)** | | **p-value** | **aHR (95%-CI)** | | **p-value** |
| **Gender** |  |  |  | |  |  | |  |
| Male | 62.3 (175±0/281) | 62.3 (175±0/281) | *** | | *** | *** | | *** |
| Female | 37.7 (106±0/281) | 37.7 (106±0/281) | *** | | *** | *** | | *** |
| **Comorbidities**** |  |  |  | |  |  | |  |
| Hypertension | 65.5 (184±1/281) | 65.4 (184±5/281) | *** | | *** | *** | | *** |
| Chronic heart failure | 9.3 (26±1/281) | 7.8 (22±4/281) | *** | | *** | *** | | *** |
| Coronary heart disease | 20.4 (57±2/281) | 18.8 (53±4/281) | *** | | *** | *** | | *** |
| Other cardiovascular disease | 29.5 (83±1/281) | 31.3 (88±6/281) | 1.07 (0.63-1.84) | | 0.796 | 1.02 (0.57-1.84) | | 0.938 |
| Diabetes mellitus type 2 | 33.3 (93±2/281) | 32.8 (92±5/281) | *** | | *** | *** | | *** |
| Chronic pulmonary disease | 25.0 (70±1/281) | 23.4 (66±5/281) | *** | | *** | *** | | *** |
| Chronic kidney disease | 13.8 (39±1/281) | 13.6 (38±4/281) | *** | | *** | *** | | *** |
| Oncological disease | 15.7 (44±1/281) | 12.9 (36±4/281) | *** | | *** | *** | | *** |
| Chronic liver disease | 1.7 (5±1/281) | 1.4 (4±2/281) | 1.53 (0.24-9.74) | | 0.653 | 1.76 (0.27-11.38) | | 0.773 |
| **BMI** |  |  |  | |  |  | |  |
| < 18,5 kg/m^2^ | 3.0 (8±2/281) | 2.3 (7±2/281) | *** | | *** | *** | | *** |
| 18,5 - 24,9 kg/m^2^ | 26.1 (73±5/281) | 25.7 (72±8/281) | *** | | *** | *** | | *** |
| 25 - 29,9 kg/m^2^ | 33.1 (93±5/281) | 35.3 (99±8/281) | *** | | *** | *** | | *** |
| > 30 kg/m^2^ | 37.8 (106±5/281) | 36.6 (103±7/281) | *** | | *** | *** | | *** |
| **Smoking history** |  |  |  | |  |  | |  |
| Active smoker | 20.6 (58±5/281) | 18.4 (52±5/281) | 0.77 (0.39-1.52) | | 0.449 | 0.74 (0.37-1.49) | | 0.728 |
| Former smoker | 24.4 (69±5/281) | 19.5 (55±6/281) | 0.89 (0.40-1.96) | | 0.769 | 0.84 (0.37-1.92) | | 0.773 |
| Non-smoker | 55.0 (154±7/281) | 62.1 (174±8/281) | Ref. | | Ref. | Ref. | | Ref. |
| **Pre-existing immunosuppressive therapy**** |  |  |  | |  |  | |  |
| Immunosuppressive therapy | 7.5 (21±1/281) | 9.7 (27±5/281) | 1.79 (0.83-3.89) | | 0.147 | 1.81 (0.82-3.95) | | 0.443 |
| **Course of SARS-CoV-2 infection** |  |  |  | |  |  | |  |
| Fatal outcome | 12.1 (34±0/281) | 18.6 (52±4/281) |  | |  |  | |  |
| ***Phase* at first positive SARS-CoV-2 detection*** |  |  |  | |  |  | |  |
| Uncomplicated phase | 34.9 (98±0/281) | 34.9 (98±0/281) | *** | | *** | *** | | *** |
| Complicated phase | 65.1 (183±0/281) | 65.1 (183±0/281) | *** | | *** | *** | | *** |
| Critical phase | 0.0 (0±0/281) | 0.0 (0±0/281) | *** | | *** | *** | | *** |
| Recovery phase | 0.0 (0±0/281) | 0.0 (0±0/281) | *** | | *** | *** | | *** |
| Dead | 0.0 (0±0/281) | 0.0 (0±0/281) | *** | | *** | *** | | *** |
| ***COVID-19 treatment***** |  |  |  | |  |  | |  |
| Remdesivir | 100.0 (281±0/281) | 0.0 (0±0/281) | 0.64 (0.40-1.02) | | 0.062 | 0.62 (0.38-1.00) | | 0.443 |
| Steroids > 0.5 mg/kg prednisolone equivalents  in the course of disease | 45.6 (128±2/281) | 42.6 (120±6/281) | *** | | *** | *** | | *** |
| Convalescent plasma | 2.5 (7±0/281) | 2.0 (6±2/281) | 1.53 (0.39-5.94) | | 0.542 | 1.36 (0.33-5.54) | | 0.773 |
| Other COVID-19 therapy | 1.1 (3±0/281) | 1.5 (4±2/281) | n.a. | | n.a. | n.a. | | n.a. |
| ***Anticoagulants*** |  |  |  | |  |  | |  |
| Therapeutic anticoagulation | 36.1 (101±1/281) | 41.4 (116±7/281) | 0.72 (0.37-1.42) | | 0.348 | 0.63 (0.31-1.27) | | 0.462 |
| Prophylactic anticoagulation | 44.3 (124±1/281) | 48.0 (135±7/281) | 0.64 (0.32-1.28) | | 0.212 | 0.58 (0.28-1.19) | | 0.443 |
| No anticoagulation | 19.6 (55±1/281) | 10.6 (30±3/281) | Ref. | | Ref. | Ref. | | Ref. |
| **Time of first positive SARS-CoV-2 detection** |  |  |  | |  |  | |  |
| First phase of COVID-19 pandemic | 17.4 (49±0/281) | 16.7 (47±2/281) | *** | | *** | *** | | *** |
| Second phase of COVID-19 pandemic | 73.3 (206±0/281) | 74.5 (209±4/281) | *** | | *** | *** | | *** |
| Third phase of COVID-19 pandemic | 9.2 (26±0/281) | 8.8 (25±4/281) | *** | | *** | *** | | *** |

Data are shown after multiple imputation, selection of patients aged 66-85 years undergoing the complicated phase of disease and matching by gender, phase at first positive SARS-CoV-2 detection, hypertension, chronic heart failure, coronary heart disease, diabetes mellitus type 2, chronic pulmonary disease, chronic kidney disease, oncological disease, body mass index (BMI) and interval of the pandemic. Distribution of factors is pooled among the imputed data and reported as mean with standard deviation rounded to integers. P value of univariable analysis after correction for multiple comparison. First phase of COVID-19 pandemic: January 2020-September 2020; second phase of COVID-19 pandemic: October 2020-Febuary 2021; third phase of COVID-19 pandemic: March 2021-December 2021; other COVID-19 therapy: IL-6R inhibitors, JAK-inhibitors or IL-1R inhibitors; other cardiovascular disease: aortic stenosis, AV block, carotic arterial disease, peripheral vascular disease and arterial fibrillation. n.a.: excluded due to model quality. * According to LEOSS (Figure S1).  ** No reference indicated in binary variables. *** Matching variables excluded from regression analysis.

**Table S6** Characteristics and Cox regression model on fatal outcome of patients aged > 85 years starting RDV therapy in complicated phase and matched controls.

| **Parameter** | **Patients undergoing complicated phase*** | | |  | | | | |
| --- | --- | --- | --- | --- | --- | --- | --- | --- |
|  | **RDV cases**  **(n=32)** | **Matched controls**  **(n=32)** | **Univariate analysis** | | | | **Multivariable analysis** | |
|  | **% (no.±SD)** | **% (no.±SD)** | **HR (95%-CI)** | | **p-value** | **aHR (95%-CI)** | | **p-value** |
| **Gender** |  |  |  | |  |  | |  |
| Male | 34.4 (11±0/32) | 34.4 (11±0/32) | *** | | *** | *** | | *** |
| Female | 65.6 (21±0/32) | 65.6 (21±0/32) | *** | | *** | *** | | *** |
| **Comorbidities**** |  |  |  | |  |  | |  |
| Hypertension | 66.9 (21±1/32) | 70.5 (23±2/32) | *** | | *** | *** | | *** |
| Chronic heart failure | 6.2 (2±0/32) | 4.2 (1±1/32) | *** | | *** | *** | | *** |
| Coronary heart disease | 28.1 (9±0/32) | 19.5 (6±1/32) | *** | | *** | *** | | *** |
| Other cardiovascular disease | 40.6 (13±0/32) | 41.2 (13±2/32) | 1.20 (0.25-5.86) | | 0.829 | 1.19 (0.22-6.40) | | 0.846 |
| Diabetes mellitus type 2 | 13.3 (4±0/32) | 9.8 (3±1/32) | *** | | *** | *** | | *** |
| Chronic pulmonary disease | 15.6 (5±0/32) | 10.3 (3±1/32) | *** | | *** | *** | | *** |
| Chronic kidney disease | 21.9 (7±0/32) | 16.2 (5±1/32) | *** | | *** | *** | | *** |
| Oncological disease | 22.3 (7±0/32) | 15.6 (5±1/32) | *** | | *** | *** | | *** |
| Chronic liver disease | 0.0 (0±0/32) | 0.2 (0±0/32) | n.a. | | n.a. | n.a. | | n.a. |
| **BMI** |  |  |  | |  |  | |  |
| < 18,5 kg/m^2^ | 5.5 (2±1/32) | 5.8 (2±2/32) | *** | | *** | *** | | *** |
| 18,5 - 24,9 kg/m^2^ | 49.8 (16±2/32) | 52.5 (17±3/32) | *** | | *** | *** | | *** |
| 25 - 29,9 kg/m^2^ | 24.4 (8±2/32) | 21.1 (7±2/32) | *** | | *** | *** | | *** |
| > 30 kg/m^2^ | 20.3 (6±2/32) | 20.6 (7±3/32) | *** | | *** | *** | | *** |
| **Smoking history** |  |  |  | |  |  | |  |
| Active smoker | 2.8 (1±1/32) | 3.1 (1±1/32) | n.a. | | n.a. | n.a. | | n.a. |
| Former smoker | 16.1 (5±1/32) | 13.1 (4±2/32) | n.a. | | n.a. | n.a. | | n.a. |
| Non-smoker | 16.1 (5±0/32) | 9.8 (3±2/32) | Ref. | | Ref. | Ref. | | Ref. |
| **Pre-existing immunosuppressive therapy**** |  |  |  | |  |  | |  |
| Immunosuppressive therapy | 1.1 (0±0/32) | 5.0 (2±1/32) | n.a. | | n.a. | n.a. | | n.a. |
| **Course of SARS-CoV-2 infection** |  |  |  | |  |  | |  |
| Fatal outcome | 6.2 (2±0/32) | 30.2 (10±3/32) |  | |  |  | |  |
| ***Phase* at first positive SARS-CoV-2 detection*** |  |  |  | |  |  | |  |
| Uncomplicated phase | 37.5 (12±0/32) | 37.5 (12±0/32) | *** | | *** | *** | | *** |
| Complicated phase | 62.5 (20±0/32) | 62.5 (20±0/32) | *** | | *** | *** | | *** |
| Critical phase | 0.0 (0±0/32) | 0.0 (0±0/32) | *** | | *** | *** | | *** |
| Recovery phase | 0.0 (0±0/32) | 0.0 (0±0/32) | *** | | *** | *** | | *** |
| Dead | 0.0 (0±0/32) | 0.0 (0±0/32) | *** | | *** | *** | | *** |
| ***COVID-19 treatment***** |  |  |  | |  |  | |  |
| Remdesivir | 100.0 (32±0/32) | 0.0 (0±0/32) | 0.19 (0.03-1.02) | | 0.081 | 0.17 (0.03-1.01) | | 0.360 |
| Steroids > 0.5 mg/kg prednisolone equivalents  in the course of disease | 50.0 (16±0/32) | 41.4 (13±2/32) | *** | | *** | *** | | *** |
| Convalescent plasma | 0.0 (0±0/32) | 2.2 (1±1/32) | n.a. | | n.a. | n.a. | | n.a. |
| Other COVID-19 therapy | 0.0 (0±0/32) | 2.2 (1±1/32) | n.a. | | n.a. | n.a. | | n.a. |
| ***Anticoagulants*** |  |  |  | |  |  | |  |
| Therapeutic anticoagulation | 48.3 (15±1/32) | 45.8 (15±2/32) | 0.99 (0.09-10.49) | | 0.992 | 0.64 (0.05-8.68) | | 0.846 |
| Prophylactic anticoagulation | 35.6 (11±1/32) | 44.4 (14±2/32) | 0.81 (0.07-9.47) | | 0.868 | 0.54 (0.03-8.66) | | 0.846 |
| No anticoagulation | 16.1 (5±0/32) | 9.8 (3±2/32) | Ref. | | Ref. | Ref. | | Ref. |
| **Time of first positive SARS-CoV-2 detection** |  |  |  | |  |  | |  |
| First phase of COVID-19 pandemic | 15.6 (5±0/32) | 11.1 (4±2/32) | *** | | *** | *** | | *** |
| Second phase of COVID-19 pandemic | 68.8 (22±0/32) | 72.5 (23±2/32) | *** | | *** | *** | | *** |
| Third phase of COVID-19 pandemic | 15.6 (5±0/32) | 16.4 (5±1/32) | *** | | *** | *** | | *** |

Data are shown after multiple imputation, selection of patients aged > 85 years undergoing the complicated phase of disease and matching by gender, phase at first positive SARS-CoV-2 detection, hypertension, chronic heart failure, coronary heart disease, diabetes mellitus type 2, chronic pulmonary disease, chronic kidney disease, oncological disease, body mass index (BMI) and interval of the pandemic. Distribution of factors is pooled among the imputed data and reported as mean with standard deviation rounded to integers. P value of univariable analysis after correction for multiple comparison. First phase of COVID-19 pandemic: January 2020-September 2020; second phase of COVID-19 pandemic: October 2020-Febuary 2021; third phase of COVID-19 pandemic: March 2021-December 2021; other COVID-19 therapy: IL-6R inhibitors, JAK-inhibitors or IL-1R inhibitors; other cardiovascular disease: aortic stenosis, AV block, carotic arterial disease, peripheral vascular disease and arterial fibrillation. n.a.: excluded due to model quality. * According to LEOSS (Figure S1).  ** No reference indicated in binary variables. *** Matching variables excluded from regression analysis.

**Table S7** Characteristics and Cox regression model on fatal outcome of patients starting RDV therapy in complicated phase without steroid administration and matched controls.

| **Parameter** | **Patients undergoing complicated phase*** | | |  | | | | |
| --- | --- | --- | --- | --- | --- | --- | --- | --- |
|  | **RDV cases**  **(n=415)** | **Matched controls**  **(n=415)** | **Univariate analysis** | | | | **Multivariable analysis** | |
|  | **% (no.±SD)** | **% (no.±SD)** | **HR (95%-CI)** | | **p-value** | **aHR (95%-CI)** | | **p-value** |
| **Age** |  |  |  | |  |  | |  |
| 18 – 45 years | 15.4 (64±0/415) | 15.4 (64±0/415) | *** | | *** | *** | | *** |
| 46 – 65 years | 41.2 (171±0/415) | 41.2 (171±0/415) | *** | | *** | *** | | *** |
| 66 – 85 years | 39.5 (164±0/415) | 39.5 (164±0/415) | *** | | *** | *** | | *** |
| > 85 years | 3.9 (16±0/415) | 3.9 (16±0/415) | *** | | *** | *** | | *** |
| **Gender** |  |  |  | |  |  | |  |
| Male | 64.6 (268±0/415) | 64.6 (268±0/415) | *** | | *** | *** | | *** |
| Female | 35.4 (147±0/415) | 35.4 (147±0/415) | *** | | *** | *** | | *** |
| **Comorbidities**** |  |  |  | |  |  | |  |
| Hypertension | 50.3 (209±1/415) | 46.7 (194±8/415) | *** | | *** | *** | | *** |
| Chronic heart failure | 4.4 (18±1/415) | 2.6 (11±3/415) | *** | | *** | *** | | *** |
| Coronary heart disease | 10.4 (43±1/415) | 8.5 (35±5/415) | *** | | *** | *** | | *** |
| Other cardiovascular disease | 16.5 (69±2/415) | 16.0 (66±5/415) | 1.94 (1.09-3.47) | | 0.298 | 1.67 (0.88-3.17) | | 0.128 |
| Diabetes mellitus type 2 | 25.4 (106±2/415) | 25.1 (104±9/415) | *** | | *** | *** | | *** |
| Chronic pulmonary disease | 16.8 (70±1/415) | 15.8 (65±6/415) | *** | | *** | *** | | *** |
| Chronic kidney disease | 9.6 (40±1/415) | 7.1 (30±4/415) | *** | | *** | *** | | *** |
| Oncological disease | 9.7 (40±1/415) | 8.2 (34±3/415) | *** | | *** | *** | | *** |
| Chronic liver disease | 2.1 (9±1/415) | 1.1 (5±2/415) | 2.62 (0.63-10.94) | | 0.332 | 3.28 (0.75-14.34) | | 0.122 |
| **BMI** |  |  |  | |  |  | |  |
| < 18,5 kg/m^2^ | 1.7 (7±2/415) | 1.3 (5±2/415) | *** | | *** | *** | | *** |
| 18,5 - 24,9 kg/m^2^ | 20.1 (84±5/415) | 19.4 (81±6/415) | *** | | *** | *** | | *** |
| 25 - 29,9 kg/m^2^ | 33.1 (137±5/415) | 35.8 (149±10/415) | *** | | *** | *** | | *** |
| > 30 kg/m^2^ | 45.1 (187±6/415) | 43.4 (180±8/415) | *** | | *** | *** | | *** |
| **Smoking history** |  |  |  | |  |  | |  |
| Active smoker | 16.9 (70±6/415) | 16.3 (68±5/415) | 1.05 (0.49-2.24) | | 0.908 | 1.01 (0.46-2.22) | | 0.990 |
| Former smoker | 19.5 (81±5/415) | 16.4 (68±7/415) | 1.23 (0.65-2.35) | | 0.660 | 1.15 (0.58-2.26) | | 0.690 |
| Non-smoker | 63.6 (264±7/415) | 67.3 (279±11/415) | Ref. | | Ref. | Ref. | | Ref. |
| **Pre-existing immunosuppressive therapy**** |  |  |  | |  |  | |  |
| Immunosuppressive therapy | 8.2 (34±1/415) | 7.3 (30±4/415) | 1.10 (0.45-2.72) | | 0.908 | 0.90 (0.34-2.39) | | 0.833 |
| **Course of SARS-CoV-2 infection** |  |  |  | |  |  | |  |
| Fatal outcome | 7.7 (32±0/415) | 11.0 (46±5/415) |  | |  |  | |  |
| ***Phase* at first positive SARS-CoV-2 detection*** |  |  |  | |  |  | |  |
| Uncomplicated phase | 37.2 (154±1/415) | 37.2 (154±1/415) | *** | | *** | *** | | *** |
| Complicated phase | 62.8 (260±1/415) | 62.8 (260±1/415) | *** | | *** | *** | | *** |
| Critical phase | 0.0 (0±0/415) | 0.0 (0±0/415) | *** | | *** | *** | | *** |
| Recovery phase | 0.0 (0±0/415) | 0.0 (0±0/415) | *** | | *** | *** | | *** |
| Dead | 0.0 (0±0/415) | 0.0 (0±0/415) | *** | | *** | *** | | *** |
| ***COVID-19 treatment***** |  |  |  | |  |  | |  |
| Remdesivir | 100.0 (415±0/415) | 0.0 (0±0/415) | 0.69 (0.42-1.14) | | 0.332 | 0.63 (0.38-1.06) | | 0.088 |
| Steroids > 0.5 mg/kg prednisolone equivalents  in the critical phase of disease | 0.8 (3±0/397) | 0.5 (2±1/402) | *** | | *** | *** | | *** |
| Convalescent plasma | 3.4 (14±0/415) | 1.6 (7±2/415) | 2.08 (0.69-6.29) | | 0.332 | 2.70 (0.78-9.35) | | 0.124 |
| Other COVID-19 therapy | 1.2 (5±0/415) | 1.8 (7±2/415) | 3.32 (0.80-13.87) | | 0.332 | 2.80 (0.62-12.68) | | 0.191 |
| ***Anticoagulants*** |  |  |  | |  |  | |  |
| Therapeutic anticoagulation | 30.7 (127±2/415) | 28.5 (118±5/415) | 1.42 (0.73-2.75) | | 0.436 | 1.24 (0.62-2.49) | | 0.547 |
| Prophylactic anticoagulation | 46.0 (191±2/415) | 55.6 (231±7/415) | 0.60 (0.29-1.25) | | 0.332 | 0.57 (0.27-1.22) | | 0.155 |
| No anticoagulation | 23.2 (96±2/415) | 15.8 (66±6/415) | Ref. | | Ref. | Ref. | | Ref. |
| **Time of first positive SARS-CoV-2 detection** |  |  |  | |  |  | |  |
| First phase of COVID-19 pandemic | 23.6 (98±0/415) | 25.4 (106±3/415) | *** | | *** | *** | | *** |
| Second phase of COVID-19 pandemic | 67.0 (278±0/415) | 64.5 (268±5/415) | *** | | *** | *** | | *** |
| Third phase of COVID-19 pandemic | 9.4 (39±0/415) | 10.0 (42±4/415) | *** | | *** | *** | | *** |

Data are shown after multiple imputation, selection of patients undergoing the complicated phase of disease and matching by age category, gender, phase at first positive SARS-CoV-2 detection, hypertension, chronic heart failure, coronary heart disease, diabetes mellitus type 2, chronic pulmonary disease, chronic kidney disease, oncological disease, body mass index (BMI), interval of the pandemic and use of steroids in the critical phase of disease. Distribution of factors is pooled among the imputed data and reported as mean with standard deviation rounded to integers. P value of univariable analysis after correction for multiple comparison First phase of COVID-19 pandemic: January 2020-September 2020; second phase of COVID-19 pandemic: October 2020-Febuary 2021; third phase of COVID-19 pandemic: March 2021-December 2021; other COVID-19 therapy: IL-6R inhibitors, JAK-inhibitors or IL-1R inhibitors; other cardiovascular disease: aortic stenosis, AV block, carotic arterial disease, peripheral vascular disease and arterial fibrillation. n.a.: excluded due to model quality. * According to LEOSS (Figure S1). ** No reference indicated in binary variables. *** Matching variables excluded from regression analysis.

**Table S8** Characteristics and Cox regression model on fatal outcome of patients starting RDV therapy in critical phase with steroid administration and matched controls.

| **Parameter** | **Patients undergoing complicated phase*** | | |  | | | | |
| --- | --- | --- | --- | --- | --- | --- | --- | --- |
|  | **RDV cases**  **(n=98)** | **Matched controls**  **(n=98)** | **Univariate analysis** | | | | **Multivariable analysis** | |
|  | **% (no.±SD)** | **% (no.±SD)** | **HR (95%-CI)** | | **p-value** | **aHR (95%-CI)** | | **p-value** |
| **Age** |  |  |  | |  |  | |  |
| 18 – 45 years | 10.2 (10±0/98) | 10.2 (10±0/98) | *** | | *** | *** | | *** |
| 46 – 65 years | 51.0 (50±0/98) | 51.0 (50±0/98) | *** | | *** | *** | | *** |
| 66 – 85 years | 36.7 (36±0/98) | 36.7 (36±0/98) | *** | | *** | *** | | *** |
| > 85 years | 2.0 (2±0/98) | 2.0 (2±0/98) | *** | | *** | *** | | *** |
| **Gender** |  |  |  | |  |  | |  |
| Male | 74.5 (73±0/98) | 74.5 (73±0/98) | *** | | *** | *** | | *** |
| Female | 25.5 (25±0/98) | 25.5 (25±0/98) | *** | | *** | *** | | *** |
| **Comorbidities**** |  |  |  | |  |  | |  |
| Hypertension | 53.1 (52±0/98) | 60.2 (59±3/98) | *** | | *** | *** | | *** |
| Chronic heart failure | 6.8 (7±1/98) | 7.6 (7±2/98) | *** | | *** | *** | | *** |
| Coronary heart disease | 20.6 (20±1/98) | 21.5 (21±3/98) | *** | | *** | *** | | *** |
| Other cardiovascular disease | 22.2 (22±1/98) | 20.6 (20±2/98) | 1.05 (0.60-1.85) | | 0.914 | 0.98 (0.55-1.76) | | 0.944 |
| Diabetes mellitus type 2 | 39.7 (39±1/98) | 39.7 (39±2/98) | *** | | *** | *** | | *** |
| Chronic pulmonary disease | 25.4 (25±1/98) | 20.4 (20±3/98) | *** | | *** | *** | | *** |
| Chronic kidney disease | 16.0 (16±1/98) | 18.0 (18±3/98) | *** | | *** | *** | | *** |
| Oncological disease | 10.4 (10±0/98) | 10.4 (10±2/98) | *** | | *** | *** | | *** |
| Chronic liver disease | 3.4 (3±1/98) | 3.6 (4±2/98) | 1.35 (0.42-4.40) | | 0.914 | 1.21 (0.35-4.20) | | 0.766 |
| **BMI** |  |  |  | |  |  | |  |
| < 18,5 kg/m^2^ | 0.1 (0±0/98) | 0.1 (0±0/98) | *** | | *** | *** | | *** |
| 18,5 - 24,9 kg/m^2^ | 13.6 (13±2/98) | 11.6 (11±3/98) | *** | | *** | *** | | *** |
| 25 - 29,9 kg/m^2^ | 39.2 (38±2/98) | 40.0 (39±4/98) | *** | | *** | *** | | *** |
| > 30 kg/m^2^ | 47.1 (46±3/98) | 48.3 (47±3/98) | *** | | *** | *** | | *** |
| **Smoking history** |  |  |  | |  |  | |  |
| Active smoker | 30.3 (30±3/98) | 21.5 (21±3/98) | 0.96 (0.51-1.84) | | 0.914 | 1.01 (0.50-2.04) | | 0.969 |
| Former smoker | 23.3 (23±2/98) | 26.9 (26±3/98) | 0.95 (0.52-1.75) | | 0.914 | 0.95 (0.49-1.85) | | 0.887 |
| Non-smoker | 46.4 (45±3/98) | 51.6 (51±4/98) | Ref. | | Ref. | Ref. | | Ref. |
| **Pre-existing immunosuppressive therapy**** |  |  |  | |  |  | |  |
| Immunosuppressive therapy | 9.5 (9±1/98) | 11.8 (12±2/98) | 1.55 (0.80-2.30) | | 0.754 | 1.43 (0.70-2.94) | | 0.328 |
| **Course of SARS-CoV-2 infection** |  |  |  | |  |  | |  |
| Fatal outcome | 36.7 (36±0/98) | 52.9 (52±3/98) |  | |  |  | |  |
| ***Phase* at first positive SARS-CoV-2 detection*** |  |  |  | |  |  | |  |
| Uncomplicated phase | 42.9 (42±0/98) | 42.9 (42±0/98) | *** | | *** | *** | | *** |
| Complicated phase | 15.3 (15±0/98) | 15.3 (15±0/98) | *** | | *** | *** | | *** |
| Critical phase | 41.9 (41±0/98) | 41.9 (41±0/98) | *** | | *** | *** | | *** |
| Recovery phase | 0.0 (0±0/98) | 0.0 (0±0/98) | *** | | *** | *** | | *** |
| Dead | 0.0 (0±0/98) | 0.0 (0±0/98) | *** | | *** | *** | | *** |
| ***COVID-19 treatment***** |  |  |  | |  |  | |  |
| Remdesivir | 100.0 (98±0/98) | 0.0 (0±0/98) | 0.62 (0.39-0.98) | | 0.440 | 0.63 (0.39-1.02) | | 0.066 |
| Convalescent plasma | 14.3 (14±0/98) | 14.3 (14±2/98) | 1.38 (0.75-2.53) | | 0.764 | 1.29 (0.68-2.46) | | 0.442 |
| Other COVID-19 therapy | 15.3 (15±0/98) | 10.1 (10±1/98) | 0.61 (0.27-1.35) | | 0.754 | 0.67 (0.29-1.56) | | 0.361 |
| ***Anticoagulants*** |  |  |  | |  |  | |  |
| Therapeutic anticoagulation | 48.6 (48±1/98) | 61.3 (60±3/98) | 1.06 (0.41-2.73) | | 0.914 | 0.93 (0.35-2.44) | | 0.879 |
| Prophylactic anticoagulation | 42.2 (41±1/98) | 36.3 (36±3/98) | 0.73 (0.27-1.93) | | 0.914 | 0.67 (0.25-1.83) | | 0.440 |
| No anticoagulation | 9.2 (9±0/98) | 2.4 (2±2/98) | Ref. | | Ref. | Ref. | | Ref. |
| **Time of first positive SARS-CoV-2 detection** |  |  |  | |  |  | |  |
| First phase of COVID-19 pandemic | 22.4 (22±0/98) | 25.2 (25±3/98) | *** | | *** | *** | | *** |
| Second phase of COVID-19 pandemic | 68.4 (67±0/98) | 65.4 (64±2/98) | *** | | *** | *** | | *** |
| Third phase of COVID-19 pandemic | 9.2 (9±0/98) | 9.4 (9±2/98) | *** | | *** | *** | | *** |

Data are shown after multiple imputation, selection of patients undergoing the critical phase of disease and matching by age category, gender, phase at first positive SARS-CoV-2 detection, hypertension, chronic heart failure, coronary heart disease, diabetes mellitus type 2, chronic pulmonary disease, chronic kidney disease, oncological disease, body mass index (BMI) and interval of the pandemic. Distribution of factors is pooled among the imputed data and reported as mean with standard deviation rounded to integers. P value of univariable analysis after correction for multiple comparison. First phase of COVID-19 pandemic: January 2020-September 2020; second phase of COVID-19 pandemic: October 2020-Febuary 2021; third phase of COVID-19 pandemic: March 2021-December 2021; other COVID-19 therapy: IL-6R inhibitors, JAK-inhibitors or IL-1R inhibitors; other cardiovascular disease: aortic stenosis, AV block, carotic arterial disease, peripheral vascular disease and arterial fibrillation. n.a.: excluded due to model quality. * According to LEOSS (Figure S1). ** No reference indicated in binary variables. *** Matching variables excluded from regression analysis.

**Table S9** Characteristics and Cox regression model on fatal outcome of patients starting RDV therapy in critical phase without steroid administration and matched controls.

| **Parameter** | **Patients undergoing complicated phase*** | | |  | | | | |
| --- | --- | --- | --- | --- | --- | --- | --- | --- |
|  | **RDV cases**  **(n=101)** | **Matched controls**  **(n=101)** | **Univariate analysis** | | | | **Multivariable analysis** | |
|  | **% (no.±SD)** | **% (no.±SD)** | **HR (95%-CI)** | | **p-value** | **aHR (95%-CI)** | | **p-value** |
| **Age** |  |  |  | |  |  | |  |
| 18 – 45 years | 12.9 (13±0/101) | 12.9 (13±0/101) | *** | | *** | *** | | *** |
| 46 – 65 years | 47.5 (48±0/101) | 47.5 (48±0/101) | *** | | *** | *** | | *** |
| 66 – 85 years | 37.6 (38±0/101) | 37.6 (38±0/101) | *** | | *** | *** | | *** |
| > 85 years | 2.0 (2±0/101) | 2.0 (2±0/101) | *** | | *** | *** | | *** |
| **Gender** |  |  |  | |  |  | |  |
| Male | 72.3 (73±0/101) | 72.3 (73±0/101) | *** | | *** | *** | | *** |
| Female | 27.7 (28±0/101) | 27.7 (28±0/101) | *** | | *** | *** | | *** |
| **Comorbidities**** |  |  |  | |  |  | |  |
| Hypertension | 60.5 (61±1/101) | 58.7 (59±3/101) | *** | | *** | *** | | *** |
| Chronic heart failure | 6.0 (6±1/101) | 4.9 (5±1/101) | *** | | *** | *** | | *** |
| Coronary heart disease | 14.3 (14±1/101) | 12.9 (13±3/101) | *** | | *** | *** | | *** |
| Other cardiovascular disease | 8.9 (9±1/101) | 17.8 (18±3/101) | 1.90 (1.00-3.60) | | 0.275 | 1.91 (0.94-3.90) | | 0.085 |
| Diabetes mellitus type 2 | 26.2 (26±1/101) | 24.7 (25±3/101) | *** | | *** | *** | | *** |
| Chronic pulmonary disease | 14.4 (14±1/101) | 15.1 (15±2/101) | *** | | *** | *** | | *** |
| Chronic kidney disease | 8.9 (9±1/101) | 6.2 (6±1/101) | *** | | *** | *** | | *** |
| Oncological disease | 8.5 (9±1/101) | 8.8 (9±3/101) | *** | | *** | *** | | *** |
| Chronic liver disease | 3.4 (3±1/101) | 2.7 (3±1/101) | 0.51 (0.07-3.66) | | 0.925 | 0.52 (0.07-3.82) | | 0.521 |
| **BMI** |  |  |  | |  |  | |  |
| < 18,5 kg/m^2^ | 0.2 (0±0/101) | 0.1 (0±0/101) | *** | | *** | *** | | *** |
| 18,5 - 24,9 kg/m^2^ | 22.7 (23±2/101) | 17.9 (18±3/101) | *** | | *** | *** | | *** |
| 25 - 29,9 kg/m^2^ | 26.9 (27±3/101) | 29.8 (30±5/101) | *** | | *** | *** | | *** |
| > 30 kg/m^2^ | 50.1 (51±3/101) | 52.1 (53±5/101) | *** | | *** | *** | | *** |
| **Smoking history** |  |  |  | |  |  | |  |
| Active smoker | 28.3 (29±3/101) | 21.8 (22±4/101) | 0.80 (0.39-1.67) | | 0.925 | 0.87 (0.42-1.82) | | 0.715 |
| Former smoker | 13.3 (13±3/101) | 22.6 (23±3/101) | 0.83 (0.37-1.89) | | 0.925 | 0.78 (0.32-1.92) | | 0.591 |
| Non-smoker | 58.4 (59±5/101) | 55.5 (56±3/101) | Ref. | | Ref. | Ref. | | Ref. |
| **Pre-existing immunosuppressive therapy**** |  |  |  | |  |  | |  |
| Immunosuppressive therapy | 8.2 (8±1/101) | 10.5 (11±2/101) | 1.13 (0.50-2.54) | | 0.925 | 1.50 (0.60-3.77) | | 0.391 |
| **Course of SARS-CoV-2 infection** |  |  |  | |  |  | |  |
| Fatal outcome | 41.6 (42±0/101) | 43.3 (44±3/101) |  | |  |  | |  |
| ***Phase* at first positive SARS-CoV-2 detection*** |  |  |  | |  |  | |  |
| Uncomplicated phase | 33.3 (34±0/101) | 33.3 (34±0/101) | *** | | *** | *** | | *** |
| Complicated phase | 27.1 (27±0/101) | 27.1 (27±0/101) | *** | | *** | *** | | *** |
| Critical phase | 39.6 (40±0/101) | 39.6 (40±0/101) | *** | | *** | *** | | *** |
| Recovery phase | 0.0 (0±0/101) | 0.0 (0±0/101) | *** | | *** | *** | | *** |
| Dead | 0.0 (0±0/101) | 0.0 (0±0/101) | *** | | *** | *** | | *** |
| ***COVID-19 treatment***** |  |  |  | |  |  | |  |
| Remdesivir | 100.0 (101±0/101) | 0.0 (0±0/101) | 0.95 (0.60-1.52) | | 0.925 | 1.04 (0.63-1.72) | | 0.879 |
| Convalescent plasma | 10.9 (11±0/101) | 7.0 (7±2/101) | 0.54 (0.19-1.52) | | 0.822 | 0.45 (0.15-1.35) | | 0.160 |
| Other COVID-19 therapy | 12.9 (13±0/101) | 6.2 (6±2/101) | 1.04 (0.45-2.38) | | 0.926 | 1.27 (0.53-3.08) | | 0.595 |
| ***Anticoagulants*** |  |  |  | |  |  | |  |
| Therapeutic anticoagulation | 51.0 (52±1/101) | 46.8 (47±4/101) | 0.80 (0.44-1.47) | | 0.925 | 0.88 (0.46-1.67) | | 0.696 |
| Prophylactic anticoagulation | 32.8 (33±1/101) | 35.8 (36±4/101) | 0.50 (0.25-0.98) | | 0.275 | 0.50 (0.24-1.04) | | 0.068 |
| No anticoagulation | 16.2 (16±1/101) | 17.4 (18±3/101) | Ref. | | Ref. | Ref. | | Ref. |
| **Time of first positive SARS-CoV-2 detection** |  |  |  | |  |  | |  |
| First phase of COVID-19 pandemic | 39.6 (40±0/101) | 43.3 (44±2/101) | *** | | *** | *** | | *** |
| Second phase of COVID-19 pandemic | 53.5 (54±0/101) | 49.3 (50±2/101) | *** | | *** | *** | | *** |
| Third phase of COVID-19 pandemic | 6.9 (7±0/101) | 7.5 (8±1/101) | *** | | *** | *** | | *** |

Data are shown after multiple imputation, selection of patients undergoing the critical phase of disease and matching by age category, gender, phase at first positive SARS-CoV-2 detection, hypertension, chronic heart failure, coronary heart disease, diabetes mellitus type 2, chronic pulmonary disease, chronic kidney disease, oncological disease, body mass index (BMI) and interval of the pandemic. Distribution of factors is pooled among the imputed data and reported as mean with standard deviation rounded to integers. P value of univariable analysis after correction for multiple comparison. First phase of COVID-19 pandemic: January 2020-September 2020; second phase of COVID-19 pandemic: October 2020-Febuary 2021; third phase of COVID-19 pandemic: March 2021-December 2021; other COVID-19 therapy: IL-6R inhibitors, JAK-inhibitors or IL-1R inhibitors; other cardiovascular disease: aortic stenosis, AV block, carotic arterial disease, peripheral vascular disease and arterial fibrillation. n.a.: excluded due to model quality. * According to LEOSS (Figure S1). ** No reference indicated in binary variables. *** Matching variables excluded from regression analysis.

**Table S10** Characteristics of cases starting RDV therapy in uncomplicated phase and matched controls - sensitivity analysis.

| **Parameter** | **Patients undergoing complicated phase*** | | |  | | | | |
| --- | --- | --- | --- | --- | --- | --- | --- | --- |
|  | **RDV cases**  **(n=259)** | **Matched controls**  **(n=259)** | **Univariate analysis** | | | | **Multivariable analysis** | |
|  | **% (no.)** | **% (no.)** | **HR (95%-CI)** | | **p-value** | **aHR (95%-CI)** | | **p-value** |
| **Age** |  |  |  | |  |  | |  |
| 18 – 45 years | 22.0 (57/259) | 22.0 (57/259) | *** | | *** | *** | | *** |
| 46 – 65 years | 34.8 (90/259) | 34.8 (90/259) | *** | | *** | *** | | *** |
| 66 – 85 years | 37.1 (96/259) | 37.1 (96/259) | *** | | *** | *** | | *** |
| > 85 years | 6.2 (16/259) | 6.2 (16/259) | *** | | *** | *** | | *** |
| **Gender** |  |  |  | |  |  | |  |
| Male | 56.0 (145/259) | 56.0 (145/259) | *** | | *** | *** | | *** |
| Female | 44.0 (114/259) | 44.0 (114/259) | *** | | *** | *** | | *** |
| **Comorbidities**** |  |  |  | |  |  | |  |
| Hypertension | 46.6 (118/253) | 46.6 (118/253) | *** | | *** | *** | | *** |
| Chronic heart failure | 4.3 (11/254) | 3.1 (8/254) | *** | | *** | *** | | *** |
| Coronary heart disease | 13.8 (35/254) | 10.8 (27/251) | *** | | *** | *** | | *** |
| Other cardiovascular disease | 13.7 (35/255) | 16.5 (42/254) | 1.52 (0.50-4.58) | | 0.693 | 1.41 (0.30-6.50) | | 0.663 |
| Diabetes mellitus type 2 | 24.3 (61/251) | 26.1 (65/249) | *** | | *** | *** | | *** |
| Chronic pulmonary disease | 15.8 (40/253) | 16.6 (42/253) | *** | | *** | *** | | *** |
| Chronic kidney disease | 10.2 (26/254) | 11.8 (30/254) | *** | | *** | *** | | *** |
| Oncological disease | 9.8 (25/254) | 8.3 (21/254) | *** | | *** | *** | | *** |
| Chronic liver disease | 2.0 (5/254) | 2.8 (7/254) | 7.87 (2.27-27.25) | | **0.010** | n.a. | | n.a. |
| **BMI** |  |  |  | |  |  | |  |
| < 18,5 kg/m^2^ | 0.0 (0/126) | 0.0 (0/127) | *** | | *** | *** | | *** |
| 18,5 - 24,9 kg/m^2^ | 24.6 (31/126) | 22.8 (29/127) | *** | | *** | *** | | *** |
| 25 - 29,9 kg/m^2^ | 42.9 (54/126) | 49.6 (63/127) | *** | | *** | *** | | *** |
| > 30 kg/m^2^ | 32.5 (41/126) | 27.6 (35/127) | *** | | *** | *** | | *** |
| **Smoking history** |  |  |  | |  |  | |  |
| Active smoker | 22.0 (20/91) | 19.4 (21/108) | 1.29 (0.23-7.16) | | 0.973 | n.a. | | n.a. |
| Former smoker | 12.1 (11/91) | 11.1 (12/108) | 1.09 (0.11-10.38) | | 0.973 | n.a. | | n.a. |
| Non-smoker | 65.9 (60/91) | 69.4 (75/108) | Ref. | | Ref. | Ref. | | Ref. |
| **Pre-existing immunosuppressive therapy**** |  |  |  | |  |  | |  |
| Immunosuppressive therapy | 9.4 (23/244) | 11.7 (28/240) | 1.72 (0.55-5.37) | | 0.635 | 1.87 (0.53-6.56) | | 0.329 |
| **Course of SARS-CoV-2 infection** |  |  |  | |  |  | |  |
| Fatal outcome | 2.3 (6/259) | 4.6 (12/259) |  | |  |  | |  |
| ***Phase* at first positive SARS-CoV-2 detection*** |  |  |  | |  |  | |  |
| Uncomplicated phase | 100 (259/259) | 100 (259/259) | *** | | *** | *** | | *** |
| Complicated phase | 0.0 (0/259) | 0.0 (0/259) | *** | | *** | *** | | *** |
| Critical phase | 0.0 (0/259) | 0.0 (0/259) | *** | | *** | *** | | *** |
| Recovery phase | 0.0 (0/259) | 0.0 (0/259) | *** | | *** | *** | | *** |
| Dead | 0.0 (0/259) | 0.0 (0/259) | *** | | *** | *** | | *** |
| ***COVID-19 treatment***** |  |  |  | |  |  | |  |
| Remdesivir | 100 (259/259) | 0.0 (0/259) | 0.48 (0.19-1.24) | | 0.295 | 0.68 (0.20-2.30) | | 0.529 |
| Steroids > 0.5 mg/kg prednisolone equivalents  in the course of disease | 69.0 (171/248) | 71.7 (177/247) | *** | | *** | *** | | *** |
| Convalescent plasma | 1.5 (4/259) | 0.8 (2/259) | n.a. | | n.a. | n.a. | | n.a. |
| Other COVID-19 therapy | 0.4 (1/259) | 1.5 (4/259) | 5.47 (0.73-41.05) | | 0.295 | 3.22 (0.20-52.73) | | 0.413 |
| ***Anticoagulants*** |  |  |  | |  |  | |  |
| Therapeutic anticoagulation | 27.6 (70/254) | 20.7 (53/256) | 3.65 (0.96-13.87) | | 0.258 | 3.21 (0.74-13.88) | | 0.118 |
| Prophylactic anticoagulation | 46.9 (119/254) | 45.7 (117/256) | 0.97 (0.22-4.28) | | 0.973 | 0.97 (0.21-4.49) | | 0.971 |
| No anticoagulation | 25.6 (65/254) | 33.6 (86/256) | Ref. | | Ref. | Ref. | | Ref. |
| **Time of first positive SARS-CoV-2 detection** |  |  |  | |  |  | |  |
| First phase of COVID-19 pandemic | 17.0 (44/259) | 15.4 (40/259) | *** | | *** | *** | | *** |
| Second phase of COVID-19 pandemic | 57.5 (149/259) | 63.3 (164/259) | *** | | *** | *** | | *** |
| Third phase of COVID-19 pandemic | 25.5 (66/259) | 21.2 (55/259) | *** | | *** | *** | | *** |

Data of the unimputed datasets were used as sensitivity analysis. Data are shown after selection of patients undergoing the complicated phase of disease and matching by age category, gender, phase at first positive SARS-CoV-2 detection, hypertension, chronic heart failure, coronary heart disease, diabetes mellitus type 2, chronic pulmonary disease, chronic kidney disease, oncological disease, body mass index (BMI), interval of the pandemic and use of steroids in the course of disease. Distribution is indicated as mean percentages (%) and mean numbers (no.). P value of univariable analysis after correction for multiple comparison. First phase of COVID-19 pandemic: January 2020-September 2020; second phase of COVID-19 pandemic: October 2020-Febuary 2021; third phase of COVID-19 pandemic: March 2021-December 2021; other COVID-19 therapy: IL-6R inhibitors, JAK-inhibitors or IL-1R inhibitors; other cardiovascular disease: aortic stenosis, AV block, carotic arterial disease, peripheral vascular disease and arterial fibrillation. n.a.: excluded due to model quality. * According to LEOSS (Figure S1). ** No reference indicated in binary variables. *** Matching variables excluded from regression analysis.

**Table S11** Characteristics of cases starting RDV therapy in complicated phase and matched controls - sensitivity analysis.

| **Parameter** | **Patients undergoing complicated phase*** | | |  | | | | |
| --- | --- | --- | --- | --- | --- | --- | --- | --- |
|  | **RDV cases**  **(n=696)** | **Matched controls**  **(n=696)** | **Univariate analysis** | | | | **Multivariable analysis** | |
|  | **% (no.)** | **% (no.)** | **HR (95%-CI)** | | **p-value** | **aHR (95%-CI)** | | **p-value** |
| **Age** |  |  |  | |  |  | |  |
| 18 – 45 years | 15.2 (106/696) | 15.2 (106/696) | *** | | *** | *** | | *** |
| 46 – 65 years | 39.8 (277/696) | 39.8 (277/696) | *** | | *** | *** | | *** |
| 66 – 85 years | 40.4 (281/696) | 40.4 (281/696) | *** | | *** | *** | | *** |
| > 85 years | 4.6 (32/696) | 4.6 (32/696) | *** | | *** | *** | | *** |
| **Gender** |  |  |  | |  |  | |  |
| Male | 66.0 (459/696) | 66.0 (459/696) | *** | | *** | *** | | *** |
| Female | 34.0 (237/696) | 34.0 (237/696) | *** | | *** | *** | | *** |
| **Comorbidities**** |  |  |  | |  |  | |  |
| Hypertension | 50.1 (340/678) | 45.6 (308/675) | *** | | *** | *** | | *** |
| Chronic heart failure | 4.6 (31/674) | 3.7 (25/674) | *** | | *** | *** | | *** |
| Coronary heart disease | 12.9 (87/675) | 10.1 (68/674) | *** | | *** | *** | | *** |
| Other cardiovascular disease | 17.2 (116/675) | 15.7 (106/677) | 1.41 (0.96-2.06) | | 0.197 | 1.23 (0.57-2.66) | | 0.594 |
| Diabetes mellitus type 2 | 26.2 (174/663) | 23.4 (154/659) | *** | | *** | *** | | *** |
| Chronic pulmonary disease | 18.2 (122/670) | 15.4 (103/667) | *** | | *** | *** | | *** |
| Chronic kidney disease | 8.9 (60/672) | 7.8 (53/675) | *** | | *** | *** | | *** |
| Oncological disease | 11.6 (78/674) | 9.1 (61/674) | *** | | *** | *** | | *** |
| Chronic liver disease | 2.7 (18/673) | 2.2 (15/679) | 1.47 (0.63-3.42) | | 0.419 | n.a. | | n.a. |
| **BMI** |  |  |  | |  |  | |  |
| < 18,5 kg/m^2^ | 1.8 (8/458) | 1.5 (7/457) | *** | | *** | *** | | *** |
| 18,5 - 24,9 kg/m^2^ | 21.0 (96/458) | 21.0 (96/457) | *** | | *** | *** | | *** |
| 25 - 29,9 kg/m^2^ | 32.5 (149/458) | 36.1 (165/457) | *** | | *** | *** | | *** |
| > 30 kg/m^2^ | 44.8 (205/458) | 41.4 (189/457) | *** | | *** | *** | | *** |
| **Smoking history** |  |  |  | |  |  | |  |
| Active smoker | 19.8 (53/268) | 20.4 (58/285) | 1.38 (0.73-2.61) | | 0.408 | 1.01 (0.50-2.02) | | 0.983 |
| Former smoker | 21.6 (58/268) | 20.4 (58/285) | 1.54 (0.86-2.74) | | 0.240 | 1.01 (0.47-2.19) | | 0.975 |
| Non-smoker | 58.6 (157/268) | 59.3 (169/285) | Ref. | | Ref. | Ref. | | Ref. |
| **Pre-existing immunosuppressive therapy**** |  |  |  | |  |  | |  |
| Immunosuppressive therapy | 7.5 (50/667) | 11.0 (73/661) | 1.83 (1.12-2.97) | | **0.050** | 1.75 (0.82-3.76) | | 0.150 |
| **Course of SARS-CoV-2 infection** |  |  |  | |  |  | |  |
| Fatal outcome | 8.2 (57/696) | 13.9 (97/696) |  | |  |  | |  |
| ***Phase* at first positive SARS-CoV-2 detection*** |  |  |  | |  |  | |  |
| Uncomplicated phase | 38.8 (268/691) | 38.8 (268/691) | *** | | *** | *** | | *** |
| Complicated phase | 61.2 (423/691) | 61.2 (423/691) | *** | | *** | *** | | *** |
| Critical phase | 0.0 (0/691) | 0.0 (0/691) | *** | | *** | *** | | *** |
| Recovery phase | 0.0 (0/691) | 0.0 (0/691) | *** | | *** | *** | | *** |
| Dead | 0.0 (0/691) | 0.0 (0/691) | *** | | *** | *** | | *** |
| ***COVID-19 treatment***** |  |  |  | |  |  | |  |
| Remdesivir | 100.0 (696/696) | 0.0 (0/696) | 0.57 (0.42-0.79) | | **0.006** | 0.38 (0.20-0.70) | | **0.002** |
| Steroids > 0.5 mg/kg prednisolone equivalents  in the course of disease | 56.8 (369/650) | 60.5 (391/646) | *** | | *** | *** | | *** |
| Convalescent plasma | 2.4 (17/696) | 3.7 (26/696) | 2.52 (1.36-4.66) | | **0.017** | 2.31 (0.97-5.51) | | 0.058 |
| Other COVID-19 therapy | 2.4 (17/696) | 2.4 (17/696) | 1.06 (0.38-2.95) | | 0.906 | 1.66 (0.33-8.23) | | 0.535 |
| ***Anticoagulants*** |  |  |  | |  |  | |  |
| Therapeutic anticoagulation | 31.8 (217/682) | 32.4 (216/667) | 1.53 (0.92-2.53) | | 0.198 | 1.32 (0.51-3.41) | | 0.570 |
| Prophylactic anticoagulation | 50.3 (343/682) | 55.2 (368/667) | 0.70 (0.43-1.16) | | 0.244 | 0.71 (0.31-1.61) | | 0.407 |
| No anticoagulation | 17.9 (122/682) | 12.4 (83/667) | Ref. | | Ref. | Ref. | | Ref. |
| **Time of first positive SARS-CoV-2 detection** |  |  |  | |  |  | |  |
| First phase of COVID-19 pandemic | 20.0 (139/696) | 19.2 (134/696) | *** | | *** | *** | | *** |
| Second phase of COVID-19 pandemic | 67.0 (466/696) | 66.7 (464/696) | *** | | *** | *** | | *** |
| Third phase of COVID-19 pandemic | 13.1 (91/696) | 14.1 (98/696) | *** | | *** | *** | | *** |

Data of the unimputed datasets were used as sensitivity analysis. Data are shown after selection of patients undergoing the complicated phase of disease and matching by age category, gender, phase at first positive SARS-CoV-2 detection, hypertension, chronic heart failure, coronary heart disease, diabetes mellitus type 2, chronic pulmonary disease, chronic kidney disease, oncological disease, body mass index (BMI), interval of the pandemic and use of steroids in the course of disease. Distribution is indicated as mean percentages (%) and mean numbers (no.). P value of univariable analysis after correction for multiple comparison. First phase of COVID-19 pandemic: January 2020-September 2020; second phase of COVID-19 pandemic: October 2020-Febuary 2021; third phase of COVID-19 pandemic: March 2021-December 2021; other COVID-19 therapy: IL-6R inhibitors, JAK-inhibitors or IL-1R inhibitors; other cardiovascular disease: aortic stenosis, AV block, carotic arterial disease, peripheral vascular disease and arterial fibrillation. n.a.: excluded due to model quality. * According to LEOSS (Figure S1). ** No reference indicated in binary variables. *** Matching variables excluded from regression analysis.

**Table S12** Characteristics of cases starting RDV therapy in critical phase and matched controls - sensitivity analysis.

| **Parameter** | **Patients undergoing critical phase** | | |  | | | | |
| --- | --- | --- | --- | --- | --- | --- | --- | --- |
|  | **RDV cases**  **(n=214)** | **Matched controls**  **(n=214)** | **Univariate analysis** | | | | **Multivariable analysis** | |
|  | **% (no.)** | **% (no.)** | **HR (95%-CI)** | | **p-value** | **aHR (95%-CI)** | | **p-value** |
| **Age** |  |  |  | |  |  | |  |
| 18 – 45 years | 12.6 (27/214) | 12.6 (27/214) | *** | | *** | *** | | *** |
| 46 – 65 years | 49.1 (105/214) | 49.1 (105/214) | *** | | *** | *** | | *** |
| 66 – 85 years | 36.5 (78/214) | 36.5 (78/214) | *** | | *** | *** | | *** |
| > 85 years | 1.9 (4/214) | 1.9 (4/214) | *** | | *** | *** | | *** |
| **Gender** |  |  |  | |  |  | |  |
| Male | 72.9 (156/214) | 72.9 (156/214) | *** | | *** | *** | | *** |
| Female | 27.1 (58/214) | 27.1 (58/214) | *** | | *** | *** | | *** |
| **Comorbidities**** |  |  |  | |  |  | |  |
| Hypertension | 56.7 (119/210) | 57.4 (121/211) | *** | | *** | *** | | *** |
| Chronic heart failure | 5.5 (11/199) | 5.0 (10/202) | *** | | *** | *** | | *** |
| Coronary heart disease | 17.2 (35/204) | 18.0 (37/205) | *** | | *** | *** | | *** |
| Other cardiovascular disease | 14.5 (29/200) | 18.6 (38/204) | 1.42 (1.02-1.97) | | 0.124 | 0.96 (0.46-2.01) | | 0.908 |
| Diabetes mellitus type 2 | 32.3 (65/201) | 28 (56/200) | *** | | *** | *** | | *** |
| Chronic pulmonary disease | 19 (38/200) | 18.5 (37/200) | *** | | *** | *** | | *** |
| Chronic kidney disease | 11.9 (24/201) | 10.4 (21/202) | *** | | *** | *** | | *** |
| Oncological disease | 9.0 (18/201) | 9.9 (20/202) | *** | | *** | *** | | *** |
| Chronic liver disease | 4.0 (8/201) | 3.5 (7/202) | 1.07 (0.51-2.24) | | 0.951 | 0.47 (0.06-3.88) | | 0.483 |
| **BMI** |  |  |  | |  |  | |  |
| < 18,5 kg/m^2^ | 0.0 (0/142) | 0.0 (0/146) | *** | | *** | *** | | *** |
| 18,5 - 24,9 kg/m^2^ | 18.3 (26/142) | 17.8 (26/146) | *** | | *** | *** | | *** |
| 25 - 29,9 kg/m^2^ | 33.1 (47/142) | 31.5 (46/146) | *** | | *** | *** | | *** |
| > 30 kg/m^2^ | 48.6 (69/142) | 50.7 (74/146) | *** | | *** | *** | | *** |
| **Smoking history** |  |  |  | |  |  | |  |
| Active smoker | 29.3 (22/75) | 27.9 (22/79) | 0.61 (0.32-1.17) | | 0.227 | 0.66 (0.32-1.38) | | 0.271 |
| Former smoker | 17.3 (13/75) | 30.4 (24/79) | 0.98 (0.56-1.73) | | 0.951 | 1.01 (0.54-1.90) | | 0.965 |
| Non-smoker | 53.3 (40/75) | 41.8 (33/79) | Ref. | | Ref. | Ref. | | Ref. |
| **Pre-existing immunosuppressive therapy**** |  |  |  | |  |  | |  |
| Immunosuppressive therapy | 8.4 (17/203) | 12.9 (26/202) | 1.41 (0.93-2.16) | | 0.218 | 1.51 (0.73-3.09) | | 0.263 |
| **Course of SARS-CoV-2 infection** |  |  |  | |  |  | |  |
| Fatal outcome | 39.3 (84/214) | 50.9 (109/214) |  | |  |  | |  |
| ***Phase* at first positive SARS-CoV-2 detection*** |  |  |  | |  |  | |  |
| Uncomplicated phase | 38.1 (80/210) | 38.1 (80/210) | *** | | *** | *** | | *** |
| Complicated phase | 21.0 (44/210) | 21.0 (44/210) | *** | | *** | *** | | *** |
| Critical phase | 41.0 (86/210) | 41.0 (86/210) | *** | | *** | *** | | *** |
| Recovery phase | 0.0 (0/210) | 0.0 (0/210) | *** | | *** | *** | | *** |
| Dead | 0.0 (0/210) | 0.0 (0/210) | *** | | *** | *** | | *** |
| ***COVID-19 treatment***** |  |  |  | |  |  | |  |
| Remdesivir | 100.0 (214/214) | 0.0 (0/214) | 0.71 (0.54-0.93) | | 0.068 | 0.91 (0.54-1.53) | | 0.728 |
| Steroids > 0.5 mg/kg prednisolone equivalents  in the course of disease | 45.0 (86/191) | 45.4 (89/196) | *** | | *** | *** | | *** |
| Convalescent plasma | 11.7 (25/214) | 8.4 (18/214) | 0.76 (0.46-1.27) | | 0.366 | 0.85 (0.34-2.10) | | 0.721 |
| Other COVID-19 therapy | 14 (30/214) | 7.9 (17/214) | 0.73 (0.44-1.20) | | 0.304 | 0.80 (0.32-2.01) | | 0.631 |
| ***Anticoagulants*** |  |  |  | |  |  | |  |
| Therapeutic anticoagulation | 49.0 (101/206) | 52.9 (109/206) | 0.71 (0.47-1.07) | | 0.218 | 0.70 (0.36-1.37) | | 0.300 |
| Prophylactic anticoagulation | 38.8 (80/206) | 37.4 (77/206) | 0.46 (0.29-0.72) | | **0.007** | 0.28 (0.13-0.63) | | **0.002** |
| No anticoagulation | 12.1 (25/206) | 9.7 (20/206) | Ref. | | Ref. | Ref. | | Ref. |
| **Time of first positive SARS-CoV-2 detection** |  |  |  | |  |  | |  |
| First phase of COVID-19 pandemic | 30.4 (65/214) | 33.6 (72/214) | *** | | *** | *** | | *** |
| Second phase of COVID-19 pandemic | 61.2 (131/214) | 56.5 (121/214) | *** | | *** | *** | | *** |
| Third phase of COVID-19 pandemic | 8.4 (18/214) | 9.8 (21/214) | *** | | *** | *** | | *** |

Data of the unimputed datasets were used as sensitivity analysis. Data are shown after selection of patients undergoing the critical phase of disease and matching by age category, gender, phase at first positive SARS-CoV-2 detection, hypertension, chronic heart failure, coronary heart disease, diabetes mellitus type 2, chronic pulmonary disease, chronic kidney disease, oncological disease, body mass index (BMI), interval of the pandemic and use of steroids in the course of disease. Distribution is indicated as mean percentages (%) and mean numbers (no.). P value of univariable analysis after correction for multiple comparison. First phase of COVID-19 pandemic: January 2020-September 2020; second phase of COVID-19 pandemic: October 2020-Febuary 2021; third phase of COVID-19 pandemic: March 2021-December 2021; other COVID-19 therapy: IL-6R inhibitors, JAK-inhibitors or IL-1R inhibitors; other cardiovascular disease: aortic stenosis, AV block, carotic arterial disease, peripheral vascular disease and arterial fibrillation. n.a.: excluded due to model quality. * According to LEOSS (Figure S1). ** No reference indicated in binary variables. *** Matching variables excluded from regression analysis.

**Table S13** Missing analyses**.**

| **Parameter** | **Missing values % (no.)** | **Test on MCAR-hypothesis (p-value)** |
| --- | --- | --- |
| Hypertension | 4.2 (409/9,687) | 0.577 |
| Chronic heart failure | 4.6 (441/9,687) | 0.692 |
| Coronary heart disease | 4.5 (435/9,687) | 0.833 |
| Other cardiovascular diseases | 4.6 (448/9,687) | 0.734 |
| Diabetes mellitus type 2 | 6.3 (613/9,687) | 1.000 |
| Chronic pulmonary disease | 4.4 (424/9,687) | 0.490 |
| Chronic kidney disease | 4.2 (409/9,687) | 0.837 |
| Oncological disease | 4.1 (395/9,687) | 1.000 |
| Chronic liver disease | 4.1 (394/9,687) | 1.000 |
| Body mass index | 41.8 (4054/9,687) | 0.581 |
| Smoking history | 58.6 (5681/9,687) | 0.669 |
| Pre-existing immunosuppressive therapy | 7.5 (729/9,687) | 0.996 |
| Phase at first positive SARS-CoV-2 detection | 0.7 (65/9,687) | 0.197 |
| Month of first positive SARS-CoV-2 detection | 0.1 (8/9,687) | <0.001 |
| Country of involved health care facility | 0.5 (49/9,687) | 0.999 |
| Steroids > 0.5 mg/kg prednisolone equivalents  in the course of disease | 8.6 (834/9,687) | 0.088 |
| Steroids > 0.5 mg/kg prednisolone equivalents  in uncomplicated phase | 6.7 (649/9,687) | 1.000 |
| Steroids > 0.5 mg/kg prednisolone equivalents  in complicated phase | 3.3 (319/9,687) | 0.133 |
| Steroids > 0.5 mg/kg prednisolone equivalents  in critical phase | 1.7 (161/9,687) | 0.331 |
| Anticoagulation | 3.9 (380/9,687) | 0.008 |

Missing rates and results of the test on missing completely at random (MCAR) are displayed for all variables containing missing values, which were chosen for further analyses
